# Inhomogeneous broadening in the time domain: Gauss–Lorentz, Gauss–Drude and Gauss–Debye material models

**DOI:** 10.1515/nanoph-2025-0044

**Published:** 2025-08-05

**Authors:** Ludmila J. Prokopeva, Alexander V. Kildishev

**Affiliations:** Elmore Family School of Electrical and Computer Engineering, Birck Nanotechnology Center and Purdue Quantum Science and Engineering Institute, 311308 Purdue University, West Lafayette, IN 47907, USA

**Keywords:** inhomogeneous broadening, Voigt profile, Gauss–Lorentz model, Gauss–Debye model, Gauss–Drude model, Brendel–Bormann model

## Abstract

Forty-five years after the initial attempts – first by Efimov–Khitrov in 1979, then by Brendel–Bormann in 1992 – we present a comprehensive, causal, and physically consistent framework for modeling the dielectric function with inhomogeneous (non-Lorentzian) broadening, where scattering becomes frequency- or time-dependent. This theoretical framework is based on spectral diffusion, described in the frequency domain by a complex probability density function and in the time domain by a matching characteristic function. The proposed approach accurately models the lineshapes resulting from multiple broadening mechanisms and enables the retrieval of intrinsic homogeneous linewidths as well as inhomogeneous disorder-controlled material dispersion features. To implement the new general dispersion function in time-domain Maxwell solvers, we have designed a constrained minimax-based semi-analytical approximation method (MiMOSA) that generates the shortest possible numerical stencils for a given approximation error. Application examples of exact and approximate MiMOSA models include the Gauss–Lorentz oscillator, Gauss–Debye relaxation, and Gauss–Drude conductivity. Although this study primarily focuses on the optical domain, the resulting models, which account for the Doppler shift, are equally applicable to other wave propagation phenomena in disordered dispersive media in a broad range of areas, including acoustics, magnonics, astrophysics, seismology, plasma, and quantum technologies.

## 1 Introduction

The fundamental understanding and predictive modeling of the broadening of the spectral line in optical systems require careful consideration of *homogeneous* and *inhomogeneous* mechanisms. These processes play crucial roles in determining the optical response of materials and are essential for understanding spectroscopic measurements and laser physics [1].

**Figure 1:**
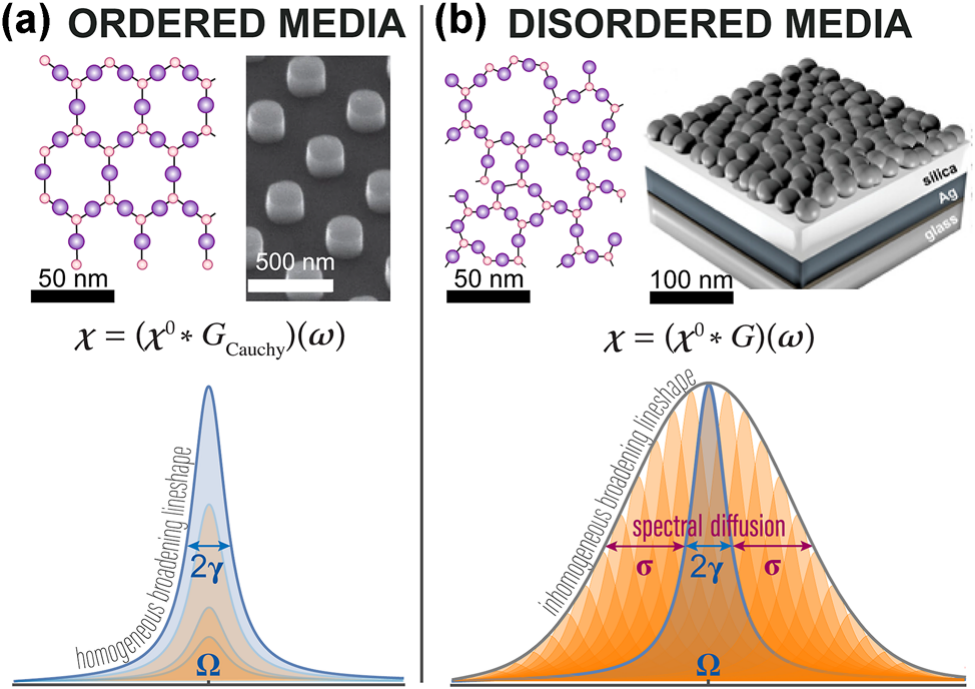
Broadening mechanisms in ordered and disordered media. (a) Ordered materials have a structured molecular or engineered arrangement, with a Lorentzian lineshape of absorption *ɛ*″(*ω*) [3]; (b) disordered materials, with lineshapes combining homogeneous (Lorentzian, *γ*) and inhomogeneous (e.g., Gaussian, *σ*) broadening, are largely inaccessible to time-domain nanophotonics due to the lack of efficient and physics-consistent models; examples: random metasurfaces, semi-continuous metal films, perovskites [4], MXenes [5], defects in oxides [6]. The peak decompositions are illustrative approximations rather than mathematically precise representations.

**Homogeneous Broadening (HB).** HB represents broadening mechanisms that affect all atoms or molecules in a system identically, arising primarily from the finite lifetime of excited states through the energy-time uncertainty principle, Δ*E*Δ*t* ∼ *ℏ*, [2]. In the statistical sense, the HB process is intimately connected to the *Cauchy distribution* (also referred to as Cauchy–Lorentz or Lorentz, Eq. (12)). This distribution arises naturally from the solution of the quantum-mechanical equation of motion for a damped oscillator, which models the atomic transition. The Cauchy distribution’s “heavy tails” (with slower decay than a Gaussian) reflect the fundamental nature of the uncertainty principle. The Cauchy distribution belongs to the class of stable distributions. Thus, in the presence of several HB mechanisms associated with the same transition frequency Ω, a sum of coherent Cauchy-distributed variates ∑_*i*_Cauchy(Ω, *γ*_*i*_) matches distribution of Cauchy(Ω, ∑_*i*_ *γ*_*i*_), preserving the location parameter Ω, as depicted in Figure 1(a). The resulting absorption spectrum follows a Lorentzian lineshape 1/(1 + *x* ^2^) with a resonant frequency Ω and a half-width-at-half-maximum (HWHM) given by *γ* = ∑_*i*_ *γ*_*i*_.

The most fundamental example of HB is a *natural line broadening* (*γ*_natural_) due to the finite lifetime of excited states. Additional HB mechanisms include *pressure broadening* (*γ*_collision_) in gases [7], where collisions interrupt the phase of atomic oscillations, and *phonon scattering* (*γ*_phonon_) in solids, which contributes to dephasing processes [8]. Using the stability of the Cauchy distribution, the total homogeneous linewidth is expressed as *γ* = *γ*_natural_ + *γ*_collision_ + *γ*_phonon_.

In the modeling sense, this simplest class of dispersion assumes that individual sources of electromagnetic response (e.g., electrons) follow identical equations of motion, with the total macroscopic model achieved via multiplication by the volume-averaged number of sources.

**Inhomogeneous Broadening (IB).** In contrast to HB, IB creates distinct subgroups of atoms or molecules with different resonant frequencies, fundamentally altering the optical response of the material system [9]. For example, in quantum dots, this phenomenon manifests itself through size distribution effects [10]. At the same time, in amorphous materials, it is caused through local structural variations modifying the electronic density of states [11], and in gas-phase systems through the IB-inducing thermal motion [12]. For example, IB plays a crucial role in modifying the optical response of quantum and nanoscale systems. In quantum cascade lasers, IB impacts emission properties, with the linewidth enhancement factor introducing phase-amplitude coupling that affects frequency comb formation [13]. At the quantum well level, studies have shown that interface roughness and well width fluctuations can lead to a significant broadening of intersubband absorption bands, with spectral hole burning experiments revealing the interplay between homogeneous and inhomogeneous contributions [14]. These IB effects have important implications for device design, as demonstrated in early work exploring intersubband scattering and coherent phenomena [15]. The fundamental understanding of IB mechanisms, presented, for example, in the work on quantum well structures [16], remains crucial to engineering and optimizing the performance of quantum and nanophotonic devices. In addition, optical materials can have intrinsic natural and fabrication defects, disorder, or amorphous structure. For example, in nanoplasmonic systems, IB arises from geometric variations in fabricated structures – even small polydispersity in parameters, such as plasmonic nanorod dimensions, can dramatically alter the optical spectra of their ensembles compared to individual elements1It is apparent that the statistical nature of structural disorder in materials plays a crucial role in determining the volume-averaged effects of IB [17]. In crystalline materials, the correlation length of the structural disorder (*ξ*) compared to the optical wavelength (*λ*) determines the strength of IB, where the volume-averaged effect scales approximately as (*ξ*/*λ*)^3^ for short-range disorder [18]. [19]. IB also occurs in natural crystals such as lithium niobate, where asymmetric infrared absorption arises from multiple anharmonic decay paths of phonon–polaritons into low-frequency phonons [20]. Finally, in photonics and plasma physics, individual carriers undergo a Doppler shift due to the Maxwellian distribution of their velocities [12]. As a result, real measured spectra deviate from the ideal Lorentzian absorption lineshape, 1/(1 + *x* ^2^), since the observed absorption peaks include two broadening mechanisms – homogeneous (*γ*) and inhomogeneous (*σ*, e.g., Gaussian), Figure 1(b). Retrieving both broadening components (*γ* and *σ*) is essential for capturing the underlying physics and tailoring the response, and requires physically consistent non-Lorentzian permittivity models.

Currently, to account for diverse IB effects with non-Lorentzian lineshapes, most ellipsometry fitting software relies on empirical *frequency-domain* approximations [21], [22]. Common examples include the pseudo-Voigt profile [23], which approximates the convolution of Lorentzian and Gaussian broadening functions (16b) with a weighted sum, and Kim’s model [24], [25], [26], which uses an empirical FD *α*-switch of the form $\gamma \left(\omega \right)=\gamma \exp\left[-\alpha {\left(\omega -\Omega \right)}^{2}/\gamma^{2}\right]$ in place of a true convolution. Additional models include spline-based approaches (e.g., Bsplines and Psemi), piecewise-smooth “stitched” absorption models (e.g., Tauc–Lorentz and Cody–Lorentz), Tanguy oscillator [27], [28], [29], [30]. Although these models are Kramers–Kronig (KK) consistent (*ɛ*′ is derived via inverse Hilbert transform of *ɛ*″), they generally lack an exact physically meaningful time-domain (TD) representation.2KK consistency does not by itself guarantee causality. Two exceptions with well-defined causal TD form are: (1) the Gaussian oscillator with characteristic (decay) function (CF) $\varphi \left(t\right)=e^{-\sigma^{2}t^{2}/2}$ (included in Table A); and (2) critical point models with decay function *φ*(*t*) ∼ e^−*γt*+(*μ*−1) ln *t*^ – a generalization of the Lorentzian (*μ* = 1) response (not included in Table A).

**Modern Experimental Techniques.** The comprehensive understanding, along with predictive and efficient numerical modeling of broadening mechanisms, have profound implications for ultra-fast laser physics [31], nanophotonic devices [32], and quantum technologies [33]. Recent advances in experimental techniques [34] continue to reveal new aspects of these fundamental processes and revolutionize our ability to study broadening mechanisms through the single-molecule [35], ultrafast [36] two-dimensional [37], and coherent multidimensional [38] spectroscopic techniques. These methods enable direct observation of individual quantum systems, provide temporal resolution of broadening dynamics, and separate homogeneous and inhomogeneous contributions. Novel spectroscopic methods [39] and advances in single-molecule detection [40] drive the development of new efficient numerical schemes that can further elucidate the complex interplay between diverse broadening phenomena and their role in areas ranging from plasma physics to emerging quantum technologies.

**Numerical Modeling in the Time Domain (TD).** The first TD models of HB dispersion were coupled with the classical finite-difference time-domain (FDTD) approximations of the Maxwell equations in the 1990s [41], [42]. Since then, multiple discretization techniques based on auxiliary differential equations (ADE) [43], [44], recursive convolution (RC) [45], [46], [47], [48], and Z-transform [49] have been developed. These methods assumed the classical Lorentz, Drude, and Debye dispersion models, where the dielectric function was given as a rational function in the FD, resulting in a set of exponential terms in the TD and ordinary differential equations with constant coefficients.

To date, efficient TD approximation schemes have been unavailable for simulations of dielectric functions that do not belong to the classical rational class. In some cases, the traditional non-Lorentzian empirical FD models are not even causal.

The present work addresses this problem for a broad class of natural and artificial materials with non-Lorentzian dispersion, where statistical averaging of individual sources results in convolved integral models. The approach begins with a causal exact description compatible with TD, where a fundamental dispersion formula is derived for an arbitrary absorption probability profile (Section 2). Section 3 expands the general formula into dispersion models for various broadening functions, yielding standard Lorentzian-type models (e.g., Lorentz, Debye, Drude) and new causal models based on Gaussian and Voigt profiles. All the models are summarized in Appendix A, Table A.

The implementation of new non-Lorentzian dispersion models in time-domain solvers (e.g., FDTD) is developed using a minimax-optimized semi-analytical approximation (MiMOSA), initially demonstrated for a causal Gaussian oscillator model [50]; here, we generalize and extend this approach to the Gauss–Lorentz, Gauss–Drude, and Gauss–Debye models (Section 3.4).

## 2 Methods

### 2.1 Probability formalism for dispersion

This section aims to formulate the material dispersion through the concept of *photon absorption probabilities* (or *broadening functions* 3The proper time-domain (TD) formulation of line broadening is not obvious [24]. Here, we define permittivity *broadening functions* *G*_*i*_(*x*) based on classical Lorentz, Debye, and Drude permittivity models grounded in the quantum mechanical differential TD equations of motion. After the *unbroadened model* is established by taking the limit *γ*_*i*_ → 0^+^, the broadening function is introduced so its Cauchy case $\left(G_{i}\left(x\right)=\gamma_{i}\pi^{-1}/\left(x^{2}+\gamma_{i}^{2}\right)\right)$ restores the classical response, and its delta-function case (*G*_*i*_(*x*) = *δ*(*x*)) reduces to the unbroadened model. Once validated, *G*_*i*_(*x*) can be any other valid *probability density function*, including Gaussian and Voigt; see similar broadening definition in Refs. [26], [51].) *G*_*i*_(*x*), enabling generalization of classical dielectric laws from Lorentz broadening to arbitrary distributions. We start with a representation of complex relative permittivity in the time and frequency domains, connected via the Fourier transform4Here *ɛ*_0_ is the vacuum permittivity, while *θ*(*x*) and *δ*(*x*) and are the Heaviside and delta functions, *ω* is the angular frequency. The hat $\left(\hat{\cdot }\right)$ and the tilde $\left(\tilde{\cdot }\right)$ denote forward and inverse Fourier-transformed functions, while the real and imaginary parts of a complex-valued function are indicated by primes, e.g., $\chi^{\prime }=R\left[\chi \right]$ and $\chi^{\prime \prime }=I\left[\chi \right]$ . The Fourier transform (FT) follows the physics convention $F\left\{f\left(t\right)\right\}=\hat{f}\left(\omega \right)=\int_{-\infty }^{\infty }f\left(t\right)e^{ı\omega t}dt$ [52].Frequency domain poles on real axis, 1/(*ω* − Ω) (Ω∈R) , are interpreted in the sense of *the Cauchy principal value* (P) ; complemented with their Hilbert transform (HT) pair, −*πδ*(*ω* − Ω), they make a Kramers–Kronig (KK)-consistent complex susceptibility term, equivalent to lossless limit according to the *Sokhotski–Plemelj theorem*

$$P\frac{1}{\omega -\Omega }-ı\pi \delta \left(\omega -\Omega \right)=\underset{\gamma \to 0^{+}}{\lim}\frac{1}{\omega -\Omega +ı\gamma }.$$

In the literature, the delta-function term is often omitted if the pole is outside the frequency range of interest. (FT, F )

(1a) $$\varepsilon \left(t\right)=\varepsilon_{\infty }\delta \left(t\right)+\frac{\sigma_{\text{e}}}{\varepsilon_{0}}\theta \left(t\right)+\underset{i}{\sum }\chi_{i}\left(t\right)\;\overset{F}{\to }$$

(1b) $$\hat{\varepsilon }\left(\omega \right)=\varepsilon_{\infty }+\frac{\sigma_{\text{e}}}{\varepsilon_{0}}\left[\pi \delta \left(\omega \right)-\frac{1}{ı\omega }\right]+\underset{i}{\sum }{\hat{\chi }}_{i}\left(\omega \right),$$

where, for generality, standard high-frequency permittivity (*ɛ*_∞_) and conductivity terms (with DC electric conductivity *σ*_e_) are assumed [53].

The dispersion terms ${\hat{\chi }}_{i}\left(\omega \right)$ are defined as ideal unbroadened susceptibilities ${\hat{\chi }}_{i}^{0}\left(\omega \right)$ convolved (broadened by) absorption probabilities *G*_*i*_(*x*), which must be valid *Probability Density Functions* (PDFs) [54], i.e., nonnengative with full probability support, *G*_*i*_(*x*) ≥ 0 and $\int_{-\infty }^{\infty }G_{i}\left(x\right)dx=1$ ,

(2) $$\begin{array}{ll} {\hat{\chi }}_{i}\left(\omega \right) & =\left({\hat{\chi }}_{i}^{0}*G_{i}\right)\left(\omega \right)\\ & =\overset{\infty }{\underset{-\infty }{\int }}{\hat{\chi }}_{i}^{0}\left(\omega -x\right)\underset{\text{PDF}}{\underset{︸}{G_{i}\left(x;\mu_{i},\sigma_{i}^{2},\ldots \right)}}\;dx. \end{array}$$

Each PDF *G*_*i*_(*x*) is parameterized by the mean (*μ*_*i*_), variance $\left(\sigma_{i}^{2}\right)$ , and/or other higher-order statistical moments and parameters. For now, we assume symmetric distributions, *G*_*i*_(−*x*) = *G*_*i*_(*x*), and zero means, *μ*_*i*_ = 0, so that the center frequency of susceptibility doesn’t change with broadening.

In the time domain, obtained via the inverse FT and applying *the convolution theorem*, Eq. (2) reads

(3) $$\chi_{i}\left(t\right)=\chi_{i}^{0}\left(t\right)\underset{\varphi_{i}\left(t\right),\text{ CF}}{\underset{︸}{\overset{\infty }{\underset{-\infty }{\int }}G_{i}\left(x\right)e^{ıxt}\;dx}},$$

where the symmetric broadening functions *G*_*i*_(*x*) contribute through its *characteristic functions* (CF) *φ*_*i*_(*t*) [54]. Standard CF properties include boundedness and zero-centered unity, |*φ*_*i*_(*t*)| ≤ 1 and *φ*_*i*_(0) = 1; moreover, if the PDF is symmetric, its CF is real-valued.

As a clear example, we reformulate the classical Lorentz oscillator using the proposed formalism

(4a) $$\chi_{\text{L}}\left(t\right)=\underset{\chi^{0}\left(t\right)}{\underset{︸}{\frac{f}{\Omega }\sin\left(\Omega t\right)\;\theta \left(t\right)}}\;\underset{\varphi_{\text{L}}\left(t\right),\text{ CF}}{\underset{︸}{e^{-\gamma t}}}\;\;\overset{F}{\to }$$

(4b) $$\begin{array}{ll} {\hat{\chi }}_{\text{L}}\left(\omega \right) & =\frac{f}{\omega_{0}^{2}-\omega^{2}-2ı\gamma \omega }\\ & =\underset{{\hat{\chi }}^{0}\left(\omega \right)}{\underset{︸}{\left(\frac{f}{\Omega^{2}-\omega^{2}}\right)}}*\underset{G_{\text{L}}\left(\omega \right),\text{ PDF}}{\underset{︸}{\left(\frac{1}{\pi }\frac{\gamma }{\omega^{2}+\gamma^{2}}\right)}}. \end{array}$$

Here *f*, *γ* are oscillator’s strength and damping parameters, while Ω and $\omega_{0}=\sqrt{\Omega^{2}-\gamma^{2}}$ are resonance and natural frequencies; *G*_L_(*x*) and *φ*_L_(*x*) are known PDF and CF of the *Cauchy–Lorentz distribution* [54], see Eq. (12); delta functions in ${\hat{\chi }}^{0}\left(\omega \right)$ are omitted for simplicity^4^.

**Figure 2:**
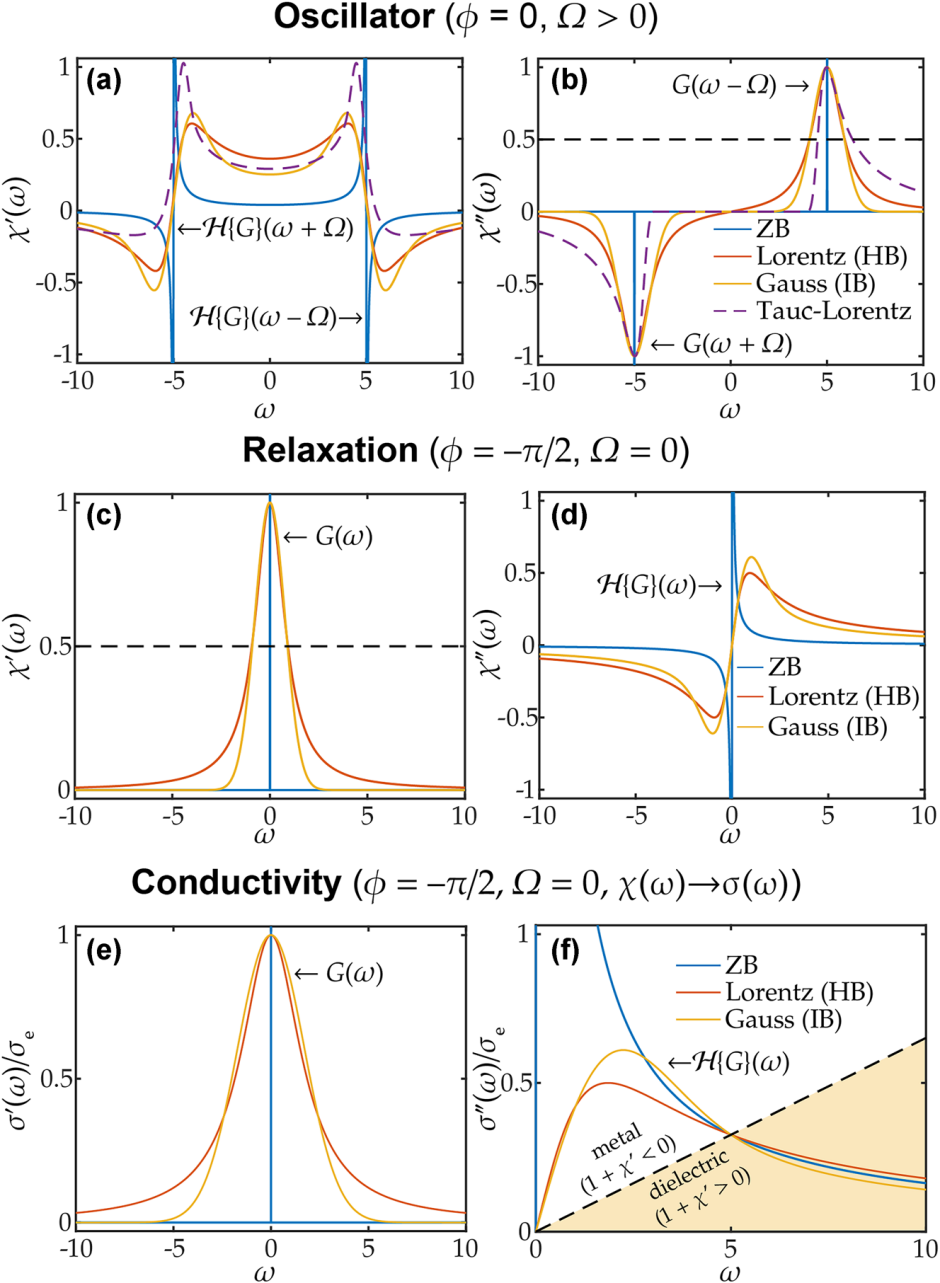
Real and imaginary parts of susceptibility $\hat{\chi }\left(\omega \right)$ (or conductivity $\hat{\sigma }\left(\omega \right)$ ) for different broadening functions: zero broadening (ZB), Lorentzian homogeneous broadening (HB) and Gaussian inhomogeneous broadening (IB) with different types of dispersion: (ab) oscillator, (cd) relaxation, (ef) conductive media, according to the newly derived formulas in this work.

Substituting the general form of the unbroadened susceptibilities $\chi_{i}^{0}\left(.\right)$ from Eq. (6), derived later in Section 3.1, into Eqs. (2) and (3) we obtain **the fundamental dispersion relation** 5The general form of unbroadened susceptibility $\chi_{i}^{0}\left(.\right)$ (Eq. (6)) is initially derived as a lossless limit of arbitrary rational susceptibility function. After the fundamental dispersion relation (5) is established, $\chi_{i}^{0}\left(.\right)$ itself becomes a trivial special case of Eq. (5) when $G_{i}\left(x\right)=\delta \left(x\right)+ı{\left(\pi x\right)}^{-1}$ and *φ*_*i*_(*t*) = 1.

(5a) $$\begin{array}{ll} \chi_{i}\left(t\right) & =\left(\chi_{i}^{0}\;\varphi_{i}\right)\left(t\right)\\ & =a_{i}\varphi_{i}\left(t\right)\sin\left(\Omega_{i}t-\phi_{i}\right)\;\theta \left(t\right)\;\overset{F}{\to } \end{array}$$

(5b) $$\begin{array}{ll} {\hat{\chi }}_{i}\left(\omega \right) & =\left({\hat{\chi }}_{i}^{0}*G_{i}\right)\left(\omega \right)\\ & =\frac{ı\pi a_{i}}{2}\left[e^{ı\phi_{i}}G_{i}\left(\omega -\Omega \right)-e^{-ı\phi_{i}}G_{i}\left(\omega +\Omega \right)\right], \end{array}$$

where $G_{i}\left(x\right)=G_{i}\left(x\right)+ıH\left\{G_{i}\left(x\right)\right\}$ is *the complex PDF* incorporating the Hilbert transform (HT, H ) of *G*_*i*_(*x*) as the imaginary part, and represents broadening, while [*a*_*i*_, *ϕ*_*i*_, Ω_*i*_] are the amplitude, phase and resonant frequency parameters of the ideal unperturbed transition (see Figure 2).

Equation (5) represents a powerful theoretical framework that generates physically consistent permittivity models for any probability distribution with known complex PDFs $G_{i}\left(x\right)$ and CFs *φ*_*i*_(*t*).6Tables of PDFs, CFs, and Hilbert transforms can be found in standard probability theory literature. In Section 3, we show how to use the general formula (5) for common broadening functions – Lorentz, Gauss, and mixed Gauss–Lorentz (Voigt), and different dispersion types – oscillator, relaxation and conductive media. A comprehensive summary of all cases and formulas, highlighting new (derived in this work) and established known models, is provided in Table A (Appendix A).

### 2.2 Analytical constraints

Time-domain modeling requires the dielectric function to be physically consistent, ensuring analyticity in the upper half-plane, causality, time-reversal symmetry (T-symmetry), Kramers–Kronig (KK) consistency, passivity and proper decay at infinity to satisfy the sum rule.

**Causality** of the total permittivity (*ɛ*(*t*) = 0, ∀*t* < 0) in Eq. (1a) is ensured as long as the unbroadened functions $\chi_{i}^{0}\left(t\right)$ are causal; e.g., general form (5a) is causal.

**T-symmetry and KK-consistency.** The real and imaginary parts of each term in (1b) satisfy the time-reversal symmetry ${\hat{\chi }}_{i}\left(-\omega \right)={\hat{\chi }}_{i}^{*}\left(\omega \right)$ and are related via the Hilbert transform (HT, H ), ensuring KK consistency,

$$H\left\{\varepsilon_{\infty }\right\}=0,\;H\left\{\pi \delta \left(\omega \right)\right\}=\omega^{-1},\;H\left\{{\hat{\chi }}_{i}^{\prime }\right\}={\hat{\chi }}_{i}^{\prime \prime }.$$

For symmetric distributions *G*_*i*_(*x*) = *G*_*i*_(−*x*), convolution (2) holds these properties, provided the unbroadened functions ${\hat{\chi }}_{i}^{0}\left(\omega \right)$ satisfy them; e.g., this holds in the general form (5b) since HT commutes with convolution and anticommutes with reflection implying $G_{i}\left(-x\right)=G_{i}^{*}\left(x\right)$ .

**Sum rules.** In ultrafast TD modeling, physically accurate high-frequency asymptotic behavior is important. As *ω* → ∞, total permittivity in Eq. (1a) should approach a free electron gas behavior: (a) ${\hat{\varepsilon }}^{\prime }\left(\omega \right)∼1-\omega_{\text{p}}^{2}/\omega^{2}$ , with (b) the imaginary part decaying faster than 1/*ω*, $\omega {\hat{\varepsilon }}^{\prime \prime }\left(\omega \right)\to 0$ , [51], [55], [56].

Condition (b) yields the sum rule $\sum_{i}a_{i}\sin\phi_{i}=\sigma_{\text{e}}\varepsilon_{0}^{-1}$ , requiring that all contributions to 1/*ω* from non-zero phase (*ϕ*_*i*_ ≠ 0) terms (e.g., conductivity, Debye, or phase-relaxed Lorentz) cancel out. This sum rule is unaffected by broadening and holds as long as satisfied for the unbroadened permittivity. Moreover, the MiMOSA approximation (Section 3.4) also conserves the sum rule (b) exactly, since $\sum_{i}\sum_{j}a_{i}^{j}\sin\phi_{i}^{j}=\sum_{i}a_{i}\sin\phi_{i}$ follows from combining conjugate pole pairs in Eq. (25) and constraint *∑*_*j*_ *B* ^*j*^ = *π* ^−1/2^.

Condition (a) in Voigt multi-term dispersion model (16) is satisfied asymptotically, in both exact and MiMOSA models, as $\varepsilon^{\prime }\left(\omega \right)-1=O\left(\omega^{-2}\right)$ , assuming *ɛ*_∞_ = 1 (often relaxed over a finite frequency ranges). The exact constant (*ω*_p_) is defined by the sum rule $\omega_{\text{p}}^{2}=\sum_{i}a_{i}\left(\Omega_{i}\cos\phi_{i}+\gamma_{i}\sin\phi_{i}\right)$ which, in general, can depend on homogeneous broadening *γ*_*i*_ (if non-zero phases *ϕ*_*i*_ are involved) but remains unaffected by inhomogeneous broadening *σ*_*i*_. For zero-phase systems (*∀i ϕ*_*i*_ = 0), the MiMOSA approximation (Section 3.4) also preserves this sum rule exactly, $\sum_{i}\sum_{j}a_{i}^{j}\left(\Omega_{i}^{j}\cos\phi_{i}^{j}+\gamma_{i}^{j}\sin\phi_{i}^{j}\right)=$ *∑*_*i*_ *a*_*i*_Ω_*i*_, see Eq. (25) for validation.

**Passivity** of the total permittivity (*ɛ*″(*ω*) ≥ 0, ∀*ω* ≥ 0) is easy to ensure in the general formulation (5b) by the passivity of individual terms, provided that all phases are zero (*ϕ*_*i*_ = 0) and the broadening functions *G*_*i*_(*x*) are *bell-shaped*.7Here, a *bell-shaped* PDF *G*(*x*) refers to a single peak PDF, symmetric about its mean (in our case, *μ* = 0): *G*(*μ* − *x*) = *G*(*μ* + *x*), ∂_*x*_ *G*(*x*) ≥ 0 for *x* < *μ* and ∂_*x*_ *G*(*x*) ≤ 0 for *x* > *μ*. When non-zero phases (*ϕ*_*i*_ ≠ 0) are present, individual terms may locally exhibit gain, compensated by other terms in the total sum. A representative class of examples are MiMOSA models in Section 3.4, where coupled oscillators with conjugate poles maintain overall passivity8In MiMOSA models, small approximation errors to passivity may arise when homogeneous broadening (*γ*_*i*_) is absent, though they decay exponentially with the number of poles. It is standard practice to restore passivity by adding a small numerical dissipation, such as homogeneous broadening (*γ*) or conductivity (*σ*_e_). (see also Figure 5 in [50]).

## 3 Results

The new probability-based dispersion relation (5) extends classical (homogeneously broadened) dispersion models – Lorentz oscillator, Debye relaxation, and Drude conductivity – to the general case of Voigt (Gauss–Lorentz) broadening and other distributions. We first derive the unbroadened case (Section 3.1), then validate the fundamental formula (5) with homogeneous (Lorentz) broadening (Section 3.2) and present new models for inhomogeneous (Gaussian and Voigt) broadening in Section 3.3.9In this section, the *single-term* susceptibility means an individual term *χ* = *χ*_*i*_, with index *i* omitted for brevity, while the *multi-term* susceptibility refers to the total sum *χ* = ∑_*i*_ *χ*_*i*_.


### 3.1 Zero broadening (ZB)

ZB represents an idealized scenario with infinitely narrow spectral lines (*G*(*x*) = *δ*(*x*)) and infinite transition lifetimes. In the class of rational functions, the general form of a single-term **unbroadened model** is derived by taking the limit *γ* → 0^+^ in the HB case (11) resulting in

(6a) $$\chi^{0}\left(t\right)=\left(\chi^{0},\varphi_{0}\right)\left(t\right)=a\sin\left(\Omega t-\phi \right)\;\theta \left(t\right)\;\overset{F}{\to }$$

(6b) $$\begin{array}{ll} {\hat{\chi }}^{0}\left(\omega \right) & =\left({\hat{\chi }}^{0}\;*\;G_{0}\right)(\omega )=\frac{a}{2}\left[\frac{e^{-ı\phi }}{\omega +\Omega }-\frac{e^{ı\phi }}{\omega -\Omega }\right]\\ & \;+\frac{ı\pi a}{2}\left[e^{ı\phi }\delta \left(\omega -\Omega \right)-e^{-ı\phi }\delta \left(\omega +\Omega \right)\right], \end{array}$$

with [*a*, *ϕ*, Ω] being the amplitude, phase and oscillation frequency parameters.

The ZB formula (6) is consistent with the fundamental dispersion equation (5), where a delta function distribution is used as the PDF,

(7) $$G_{0}\left(x\right)=\delta \left(x\right),\;G_{0}\left(x\right)=\delta \left(x\right)+\frac{ı}{\pi x},\;\varphi_{0}\left(t\right)=1,$$

and represents zero scattering *γ* = 0^+^.10The ZB case (6) represents the limit *γ* → 0^+^ of the HB case (11), under the assumption of constant amplitude *a*. When amplitude depends on *γ* (as in the Drude susceptibility), this limit yields a different result (10b). To maintain a unified formalism, broadening in the Drude case is introduced in the conductivity function: $\hat{\sigma }(\omega )={\hat{\sigma }}_{0}(\omega )\;*\;G(x)$ .


The phase parameter *ϕ* in (6) (also known as the loss angle) mixes the real and imaginary parts and allows the transition between two orthogonal cases: (*ϕ* = 0, Ω > 0) representing a classical oscillator and $\left(\phi =-\frac{\pi }{2},\Omega =0\right)$ corresponding to a relaxation in the time domain. The two cases of the ZB formula (6), along with a case for conductive media, are addressed below.

**Lossless Lorentz oscillator** (*ϕ* = 0, Ω > 0), also called the Sellmeier model [57], has quadratically decaying real part and delta functions in absorption

(8) $$\begin{array}{cc} \chi_{\text{L}}^{0}\left(t\right) & =a\sin\left(\Omega t\right)\;\theta \left(t\right)\;\overset{F}{\to }\\ {\hat{\chi }}_{\text{L}}^{0}\left(\omega \right) & =\frac{a\Omega }{\Omega^{2}-\omega^{2}}+\frac{ı\pi a}{2}\left[\delta \left(\omega -\Omega \right)-\delta \left(\omega +\Omega \right)\right]. \end{array}$$

**Lossless Debye relaxation** $\left(\phi =-\frac{\pi }{2},\Omega =0\right)$ , is simply a DC conductivity term [58]

(9) $$\chi_{\text{De}}^{0}\left(t\right)=a\theta \left(t\right)\;\overset{F}{\to }\;{\hat{\chi }}_{\text{De}}^{0}\left(\omega \right)=a\left[\pi \delta \left(\omega \right)-\frac{1}{ı\omega }\right].$$

**Lossless Drude model** $\left(\phi =-\frac{\pi }{2},\Omega =0,a=\varepsilon_{0}\omega_{\text{p}}^{2},\chi \left(.\right)\to \sigma \left(.\right)\right)$ is handled as a Debye case (9) but with a switch from susceptibility *χ*(.) to conductivity *σ*(.),11Traditionally, the Drude model is represented through susceptibility function *χ*(.) as a case of an overdamped Lorentz oscillator with zero natural frequency (*ω*_0_ = 0 or Ω = *ıγ*), where one of the two real poles is at zero and corresponds to a DC conductivity. This holds only for the HB case, but is unphysical for ZB and IB cases, while the definition through conductivity function *σ*(.) (10a) with post-conversion to *χ*(.) applies universally to all broadening cases (ZB, HB, and IB) of Eq. (5).
^,^ 12Conductivity and susceptibility functions are conventionally related through conversion formulas: $\hat{\sigma }\left(\omega \right)=-ı\omega \varepsilon_{0}\hat{\chi }\left(\omega \right)$ , *σ*(*t*) = *ɛ*_0_∂_*t*_ *χ*(*t*), $\chi \left(t\right)=\varepsilon_{0}^{-1}\int_{0}^{t}\sigma \left(\tau \right)d\tau$ (see, e.g., [53]).

(10a) $$\begin{array}{ll} \sigma_{\text{D}}^{0}\left(t\right) & =\varepsilon_{0}\omega_{\text{p}}^{2}\theta \left(t\right)\;\overset{F}{\to }\\ {\hat{\sigma }}_{\text{D}}^{0}\left(\omega \right) & =\varepsilon_{0}\omega_{\text{p}}^{2}\left[\pi \delta \left(\omega \right)-\frac{1}{ı\omega }\right], \end{array}$$

(10b) $$\chi_{\text{D}}^{0}\left(t\right)=t\omega_{\text{p}}^{2}\theta \left(t\right)\;\overset{F}{\to }\;{\hat{\chi }}_{\text{D}}^{0}\left(\omega \right)=\omega_{\text{p}}^{2}\left[-\frac{1}{\omega^{2}}-ı\pi \delta^{\prime }\left(\omega \right)\right].$$

Here *ω*_p_ is a plasma frequency – a characteristic point where the lossless Drude permittivity $\varepsilon \left(\omega \right)=1-\frac{\omega_{\text{p}}^{2}}{\omega^{2}}$ switches from metallic to dielectric behavior [59].

The delta function terms in (6b), often omitted in the literature, represent degenerate distributions of zero width and play a key role in the convolution formalism. When the ZB model (6b) is convolved with a PDF *G*(*x*), the absorption of an oscillator (*ϕ* = 0) is directly linked to the function *G*(*x*) as

$${\hat{\chi }}^{\prime \prime }\left(\omega \right)=\left({\hat{\chi }}^{0\prime \prime }*G\right)\left(\omega \right)=\frac{a\pi }{2}\left[G\left(\omega -\Omega \right)-G\left(\omega +\Omega \right)\right].$$

This is why, for example, a Gaussian distribution produces a Gaussian lineshape in the absorption. In the case of relaxation/conduction, the lineshape (of $\hat{\chi }\left(\omega \right)$ or $\hat{\sigma }\left(\omega \right)$ ) is zero-centered and is rotated by $\phi =-\frac{\pi }{2}$ from the imaginary part to the real part, e.g., ${\hat{\chi }}^{\prime }\left(\omega \right)=a\pi G\left(\omega \right)$ , as shown in Figure 2.

### 3.2 Homogeneous broadening (HB)

HB represents the natural linewidth broadening that affects all atoms or molecules equally, due to the finite lifetime *τ* = *γ* ^−1^ of excited states (uncertainty principle [2]). The general form of single-term HB dispersion, also known as **the critical point model** [60], represents an arbitrary rational function13Fourier transform of a real function *χ*(*t*) can be always approximated as a rational function of argument *s* = −*ıω* with real coefficients. We assume no multiple poles and zero limit at infinity (as the constant term *ɛ*_∞_ is detached), so partial fraction decomposition of $\hat{\chi }\left(\omega \right)$ is a sum of real poles (relaxations) and/or complex conjugate pole pairs (phase-relaxed oscillators) ${\hat{\chi }}_{i}\left(\omega \right)$ .

(11a) $$\begin{array}{cc} \chi^{\gamma }\left(t\right) & =\left(\chi^{0}\;\varphi_{\text{L}}\right)\left(t\right)\\ & =ae^{-\gamma t}\sin\left(\Omega t-\phi \right)\;\theta \left(t\right)\;\overset{F}{\to } \end{array}$$

(11b) $$\begin{array}{cc} {\hat{\chi }}^{\gamma }\left(\omega \right) & =\left({\hat{\chi }}^{0}*G_{\text{L}}\right)\left(\omega \right)\\ & =\frac{a}{2}\left[\frac{e^{-ı\phi }}{\omega +ı\gamma +\Omega }-\frac{e^{ı\phi }}{\omega +ı\gamma -\Omega }\right]. \end{array}$$

The HB case (11) can be derived by either convolving (“blurring”) the ideal unbroadened susceptibility ${\hat{\chi }}^{0}\left(\omega \right)$ in (6) with the Cauchy–Lorentz PDF *G*_L_(*x*), or substituting complex function $G_{\text{L}}\left(x\right)$ into the general formulation (5), where

(12) $$\begin{array}{cc} G_{\text{L}}\left(x;0,\gamma \right) & =\frac{1}{\pi }\frac{\gamma }{x^{2}+\gamma^{2}},\;G_{\text{L}}\left(x\right)=\frac{ı}{\pi }\frac{1}{x+ı\gamma },\\ \varphi_{\text{L}}\left(t\right) & =e^{-\gamma |t|}. \end{array}$$

As expected, the parameter substitutions (outlined in parenthesis) reduce the general HB formula (11) to the classical Lorentz [61], Debye [62], and Drude [59] dispersion models, as shown below.

**Lorentz oscillator** $\left(\phi =0,\Omega =\sqrt{\omega_{0}^{2}-\frac{\Gamma^{2}}{4}},a=\frac{f}{\Omega },\right.\left.\gamma =\frac{\Gamma }{2}\right)$ is conventionally formulated with doubled broadening Γ = 2*γ*, the natural frequency $\omega_{0}=\sqrt{\Omega^{2}+\gamma^{2}}$ instead of resonance frequency Ω, and oscillator strength *f* = *a*Ω instead of amplitude *a*,

(13) $$\begin{array}{cc} \chi_{\text{L}}^{\Gamma }\left(t\right) & =\frac{f}{\Omega }e^{-\frac{\Gamma t}{2}}\sin\left(\Omega t\right)\;\theta \left(t\right)\;\;\overset{F}{\to }\\ {\hat{\chi }}_{\text{L}}^{\Gamma }\left(\omega \right) & =\frac{f}{\omega_{0}^{2}-\omega^{2}-ı\omega \Gamma }. \end{array}$$

**Debye relaxation** $\left(\phi =-\frac{\pi }{2},\Omega =0,a=\frac{\Delta \varepsilon }{\tau },\gamma =\frac{1}{\tau }\right)$ conventionally uses parameters of relaxation time *τ* = *γ* ^−1^ and permittivity jump Δ*ɛ* = *aτ*,

(14) $$\chi_{\text{De}}^{\tau }\left(t\right)=\frac{\Delta \varepsilon }{\tau }e^{-\frac{t}{\tau }}\theta \left(t\right)\;\overset{F}{\to }\;\;{\hat{\chi }}_{\text{De}}^{\tau }\left(\omega \right)=\frac{\Delta \varepsilon }{1-ı\omega \tau }.$$

**Drude model** $\left(\phi =-\frac{\pi }{2},\Omega =0,a=\varepsilon_{0}\omega_{\text{p}}^{2},\chi \left(.\right)\to \sigma \left(.\right)\right)$ is classically parameterized by the plasma frequency *ω*_p_ and broadening *γ* and is obtained as the Debye case of the conductivity function^11^

(15a) $$\sigma_{\text{D}}^{\gamma }\left(t\right)=\varepsilon_{0}\omega_{\text{p}}^{2}e^{-\gamma t}\theta \left(t\right)\;\overset{F}{\to }\;\;{\hat{\sigma }}_{\text{D}}^{\gamma }\left(\omega \right)=\frac{\varepsilon_{0}\omega_{\text{p}}^{2}}{\gamma -ı\omega },$$

(15b) $$\begin{array}{cc} \chi_{\text{D}}^{\gamma }\left(t\right) & =\frac{\omega_{\text{p}}^{2}}{\gamma }\left(1-e^{-\gamma t}\right)\theta \left(t\right)\;\overset{F}{\to }\\ {\hat{\chi }}_{\text{D}}^{\gamma }\left(\omega \right) & =-\frac{\omega_{\text{p}}^{2}}{ı\omega \gamma +\omega^{2}}+\frac{\pi \omega_{\text{p}}^{2}}{\gamma }\delta \left(\omega \right). \end{array}$$

In the Drude case, convolution with unbroadened susceptibility (10b) is unphysical but valid for its unbroadened conductivity function (10a), with *χ*(.) restored from *σ*(.) afterward.^12^

### 3.3 Inhomogeneous broadening (IB)

IB arises from statistical distribution of microscopic resonant frequencies Ω affected by local environmental variations and *the Doppler shift*.14Gaussian IB essentially represents the Doppler shift effect, when the particles “see” the wavelength differently according to the Maxwellian distribution of the velocities [12]. As a result, the observed spectral broadening deviates from the ideal Lorentzian *lineshape* to a mix of both – natural lifetime-based (HB) defined by *γ* and statistical (e.g., Gaussian) broadening defined by variance *σ* ^2^ (Figure 1), leading to the general **Gauss–Lorentz model**

(16a) $$\begin{array}{cc} \chi^{\gamma ,\sigma }\left(t\right) & =\left(\chi^{0}\varphi_{\text{L}}\varphi_{\text{G}}\right)\left(t\right)\\ & =ae^{-\gamma t-\frac{\sigma^{2}t^{2}}{2}}\sin\left(\Omega t-\phi \right)\;\theta \left(t\right)\;\overset{F}{\to }\; \end{array}$$

(16b) $$\begin{array}{cc} {\hat{\chi }}^{\gamma ,\sigma }\left(\omega \right) & =\left({\hat{\chi }}^{0}*G_{\text{L}}*G_{\text{G}}\right)\left(\omega \right)\\ & =\frac{ıa\sqrt{\pi }}{2\sigma \sqrt{2}}\left[\begin{array}{cc} & e^{ı\phi }w\left(\frac{\omega +ı\gamma -\Omega }{\sigma \sqrt{2}}\right)\\ & \;-e^{-ı\phi }w\left(\frac{\omega +ı\gamma +\Omega }{\sigma \sqrt{2}}\right) \end{array}\right], \end{array}$$

where *w*(*z*) is the Faddeeva (Kramp) function [63].

The IB formula (16) is obtained as a convolution of the unbroadened response ${\hat{\chi }}^{0}\left(\omega \right)$ with both the Cauchy PDF (*G*_L_, see Eq. (12)) and the Gaussian PDF (*G*_G_) defined by

(17) $$\begin{array}{cc} G_{\text{G}}\left(x;0,\sigma^{2}\right) & =\frac{e^{-\frac{x^{2}}{2\sigma^{2}}}}{\sigma \sqrt{2\pi }},\;G_{\text{G}}\left(x\right)=\frac{w\left(\frac{x}{\sigma \sqrt{2}}\right)}{\sigma \sqrt{2\pi }},\\ \varphi_{\text{G}}\left(t\right) & =e^{-\sigma^{2}t^{2}/2}, \end{array}$$

corresponding to the time-dependent scattering *γ*(*t*) = *σ* ^2^ *t*/2. In the presence of multiple broadening mechanisms, the probability theory for the sum of random variables dictates that the PDFs are convolved, while their CFs are multiplied,15Both operations (∗ and ⋅) are commutative and associative, e.g., $\left(\chi^{0}*G_{1}\right)*G_{2}=\chi^{0}*\left(G_{1}*G_{2}\right)=\chi^{0}*\left(G_{2}*G_{1}\right)$ , (under suitable integrability conditions). and so the IB formula (16) can also be obtained from the general formula (5) using the Gauss–Lorentz (Voigt) PDF/CF

(18) $$\begin{array}{cc} G_{\text{GL}}\left(x;\gamma ,\sigma \right) & =\left(G_{\text{L}}*G_{\text{G}}\right)\left(x\right)\\ & =\frac{1}{\sigma \sqrt{2\pi }}Rw\left(\frac{x+ı\gamma }{\sigma \sqrt{2}}\right),\\ G_{\text{GL}}\left(x\right) & =\frac{1}{\sigma \sqrt{2\pi }}w\left(\frac{x+ı\gamma }{\sigma \sqrt{2}}\right),\\ \varphi_{\text{GL}}\left(t\right) & =\left(\varphi_{\text{L}}\;\varphi_{\text{G}}\right)\left(t\right)=e^{-\gamma |t|}e^{-\sigma^{2}t^{2}/2}, \end{array}$$

where the scattering function has both – the constant and the linear correction terms, *γ*(*t*) = *γ* + *σ* ^2^ *t*/2.16Higher orders terms in the scattering function *γ*(*t*) are also possible with other (non-Gaussian) distributions.


The new Voigt formula is consistent with all limiting cases: *σ* → 0^+^ gives classical Lorentzian models (11), *γ* → 0^+^ gives pure Gaussian lineshape typical for strong disorder, while *σ*, *γ* → 0^+^ gives the ZB case (6). These transitions are easy to see through the general formula (5) and distributions equations (7), (12), (17) and (18).17Note that in the Gaussian limit (*γ* = 0^+^) of all equations with IB parameter *σ*, the arguments of the Faddeeva function *w*(*x*) become real, so that the real and imaginary parts of *w*(*x*) can be separated as $w\left(x\right)=e^{-x^{2}}+\frac{2ı}{\sqrt{\pi }}F\left(x\right)$ , where *F*(*x*) is the Dawson function. In the Lorentzian limit (*σ* = 0^+^), large argument formulas are useful: $w\left(z\right)\approx \frac{ı}{z\sqrt{\pi }}$ or $\text{erfc}\left(z\right)=e^{-z^{2}}w\left(ız\right)\approx \frac{e^{-z^{2}}}{z\sqrt{\pi }}$ .


As before, we simplify the general IB formula (16) for the oscillator, relaxation, and conductivity cases, specifying the corresponding parameter substitutions.

**Gauss-Lorentz oscillator** (*ϕ* = 0, $\Omega =\sqrt{\omega_{0}^{2}-\frac{\Gamma^{2}}{4}}$ , $a=\frac{f}{\Omega }$ , $\gamma =\frac{\Gamma }{2})$ with strength *f*, natural frequency *ω*_0_ and two (HB and IB) broadening parameters Γ, *σ* reads

(19) $$\begin{array}{cc} \chi_{\text{L}}^{\Gamma ,\sigma }\left(t\right) & =\frac{f}{\Omega }e^{-\frac{\Gamma t}{2}-\frac{\sigma^{2}t^{2}}{2}}\sin\left(\Omega t\right)\;\theta \left(t\right)\;\overset{F}{\to }\\ {\hat{\chi }}_{\text{L}}^{\Gamma ,\sigma }\left(\omega \right) & =\frac{ıf\sqrt{\pi }}{2\sqrt{2}\sigma \Omega }\left[\begin{array}{cc} & w\left(\frac{\omega +\frac{ı\Gamma }{2}-\Omega }{\sigma \sqrt{2}}\right)\\ & \;-w\left(\frac{\omega +\frac{ı\Gamma }{2}+\Omega }{\sigma \sqrt{2}}\right) \end{array}\right]. \end{array}$$

The first causal Gaussian oscillator model (Γ = 0^+^) was derived in 2006 [64] using a causality tip from [65]. The first attempts to formulate the Gauss–Lorentz model date back to the late 1970s [66], with a later reproduction [67] usually referred to as the Brendel-Bormann (BB)-model. Unfortunately, the non-causal BB model, incompatible with TD, remains broadly adopted by experimentalists to fit material responses to infra-red light, e.g., [68], [69], [70]. Causal corrections with logarithmic terms, rational approximations, and ongoing discussions of the physical validity of the BB model can be found in Refs. [71], [72], [73].

Our new Gauss–Lorentz (GL) model (19) fixes all the issues with the previous BB formulation [67] (see the details in Appendix B). When *σ* → 0^+^, the GL formula gives the classical Lorentz model with 1/(1 + *x* ^2^) absorption lineshape, (13). The case of Γ → 0^+^ gives a causal Gaussian oscillator with exp[−*x* ^2^ ln2] absorption lineshape^17^ [64], while the mixed case (Γ, *σ* > 0) yields the Voigt profile – a new causal formulation with a time-dependent scattering function *γ*(*t*) = *γ* + *σ* ^2^ *t*/2, first mentioned by Kim et al. [24], [25], and consistent with [74].

**Gauss–Debye relaxation** $(\phi =-\frac{\pi }{2}$ , Ω = 0, $a=\frac{\Delta \varepsilon }{\tau }$ , $\gamma =\frac{1}{\tau })$ with the IB parameter *σ* additionally to the standard relaxation time *τ* and permittivity jump Δ*ɛ* reads

(20) $$\begin{array}{cc} \chi_{\text{De}}^{\tau ,\sigma }\left(t\right) & =\frac{\Delta \varepsilon }{\tau }e^{-\frac{t}{\tau }-\frac{\sigma^{2}t^{2}}{2}}\theta \left(t\right)\;\overset{F}{\to }\\ {\hat{\chi }}_{\text{De}}^{\tau ,\sigma }\left(\omega \right) & =\frac{\Delta \varepsilon \sqrt{\pi }}{\sigma \tau \sqrt{2}}\;w\left(\frac{\omega +ı\tau^{-1}}{\sigma \sqrt{2}}\right). \end{array}$$

The limits *σ* → 0^+^ and *γ* → 0^+^ give the classical Debye (14) and a new Gauss relaxation model, respectively.^17^

The Gauss–Debye model (20) has not been shown in the literature. Known generalizations to the Debye relaxation – the Cole–Cole, Cole–Davidson, and Havriliak–Negami models [75], [76] – remain inaccessible to efficient TD simulations, and will be addressed in future work.

**Gauss-Drude model** $(\phi =-\frac{\pi }{2}$ , Ω = 0, $a=\varepsilon_{0}\omega_{\text{p}}^{2}$ , *χ*(.) → *σ*(.)) with IB broadening parameter *σ* additionally to the plasma frequency *ω*_p_ and HB *γ* reads

(21a) $$\begin{array}{cc} \sigma_{\text{D}}^{\gamma ,\sigma }\left(t\right) & =\varepsilon_{0}\omega_{\text{p}}^{2}e^{-\gamma t-\frac{\sigma^{2}t^{2}}{2}}\theta \left(t\right)\;\overset{F}{\to }\\ {\hat{\sigma }}_{\text{D}}^{\gamma ,\sigma }\left(\omega \right) & =\varepsilon_{0}\frac{\omega_{\text{p}}^{2}\sqrt{\pi }}{\sigma \sqrt{2}}\;w\left(\frac{\omega +ı\gamma }{\sigma \sqrt{2}}\right), \end{array}$$

(21b) $$\begin{array}{cc} \chi_{\text{D}}^{\gamma ,\sigma }\left(t\right) & =\frac{\omega_{\text{p}}^{2}\sqrt{\pi }}{\sigma \sqrt{2}}e^{\frac{\gamma^{2}}{2\sigma^{2}}}\\ & \;\times \left[\begin{array}{cc} & \text{erfc}\left(\frac{\gamma }{\sigma \sqrt{2}}\right)\\ & \;-\text{erfc}\left(\frac{\gamma +\sigma^{2}t}{\sigma \sqrt{2}}\right) \end{array}\right]\theta \left(t\right)\;\overset{F}{\to }\\ {\hat{\chi }}_{\text{D}}^{\gamma ,\sigma }\left(\omega \right) & =\frac{\omega_{\text{p}}^{2}\sqrt{\pi }}{\sigma \sqrt{2}}\left[\begin{array}{c} \pi w\left(\frac{ı\gamma }{\sigma \sqrt{2}}\right)\;\delta \left(\omega \right)\\ -\frac{1}{ı\omega }w\left(\frac{\omega +ı\gamma }{\sigma \sqrt{2}}\right) \end{array}\right]. \end{array}$$

The limits *σ* → 0^+^ and *γ* → 0^+^ give the classical Drude model (15b) and a new Gauss conductive model, respectively.^17^ The derivation is based on the broadening formalism (Section 2) for the conductivity function. Comparing classical *Drude model* (15b) to the new *Gauss conductivity model* (21b), we observe lineshape change and find that disordered analogue of classic conductivity $\varepsilon_{0}\omega_{\text{p}}^{2}/\gamma$ , is $\varepsilon_{0}\omega_{\text{p}}^{2}\sqrt{\pi }/\left(\sigma \sqrt{2}\right)$ . In the TD, linear argument of the exponential decay in *σ*(*t*) becomes quadratic, and in *χ*(*t*) changes to a complementary error function (erfc).

Corrections to the classical Drude model have been widely studied, including empirical frequency-domain formulations with fractional derivatives, effective mass parameter, and modified scattering functions [77]. However, the causal Gauss–Drude model introduced here has never been presented.

Figure 2 illustrates the final Voigt formula (16) for three cases of broadening – ZB, HB (*σ* = 0^+^) and IB (*γ* = 0^+^) for three types of dispersion: oscillator, relaxation, and conductive media. HB and IB curves are matched at the peak maximum and full-width-half-maximum (FWHM) of the real (for relaxation) or imaginary (for oscillator) parts, demonstrating the deviation of the “heavy-tail” Lorentzian lineshape $\frac{1}{1+x^{2}}$ from the Gaussian lineshape $e^{-x^{2}\;\ln\;2}$ . For conductive media, the DC conductivity ( $\hat{\sigma }$ (0)) and a plasma crossover point $\left(\omega =\omega_{\text{p}}\right)$ are matched.

These plots effectively illustrate the physical interpretation of the complex PDF G(x)=G(x)+ıH{G(x)} . For an oscillator with zero phase (*ϕ* = 0), the real part of G(x) defines the absorption spectrum $\left({\hat{\chi }}^{\prime \prime }\left(\omega \right)\right)$ , producing symmetric peaks *G*(*ω* ± Ω), while the imaginary part generates KK-consistent terms H{G}(ω±Ω) in ${\hat{\chi }}^{\prime }\left(\omega \right)$ . When the phase *ϕ* is nonzero, the real and imaginary parts of the susceptibility become mixed. In the case of relaxation (*ϕ* = −*π*/2), ${\hat{\chi }}^{\prime }$ and ${\hat{\chi }}^{\prime \prime }$ are effectively swapped, and the two resonant peaks coalesce into a single peak. The conductive case is identical to the relaxation case, with the susceptibility function $\hat{\chi }\left(\omega \right)$ replaced by the conductivity function $\hat{\sigma }\left(\omega \right)$ .

### 3.4 Minimax approximation (MiMOSA)

When the broadening function *G*(*x*) is non-Lorentzian, the general dispersion formula (5) falls outside of the *class of the rational functions* of argument *s* = −*ıω*, and cannot be immediately translated into auxiliary differential equations, making it challenging to construct short discretization stencils and coupling to time-domain solvers, such as FDTD. The solution for efficient TD implementation of non-Lorentzian dispersion was first developed for a pure Gaussian oscillator [50], and employs *minimax optimization* to generate the shortest possible time stencil for a given error (MiMOSA).

Derivation of MiMOSA (Mini-max optimized semianalytical approximation) models for a general dispersion formula (5) starts with a minimax rational approximation18Constraints are imposed on parity, pole positioning, and the sum rule, with the objective function optimized using the *minimax criterion* on the real axis. Restoring the complex-valued approximation with the Hilbert transform and then analytically continuing the approximation to the upper half-space gives the complex PDF approximation. of the complex PDF $G\left(x\right)\;=\;G\left(x\right)\;+\;ıH\left\{G,\left(x\right)\right\}$ ,

(22) $$\begin{array}{ll} & \sigma \sqrt{2\pi }\;G\left(z\sigma \sqrt{2};\gamma =0\right)=w\left(z\right)\\ & \approx w^{n}\left(z\right)=\overset{n}{\underset{j=1}{\sum }}\frac{B^{j}}{\left(-ız\right)-C^{j}}. \end{array}$$

**Figure 3:**
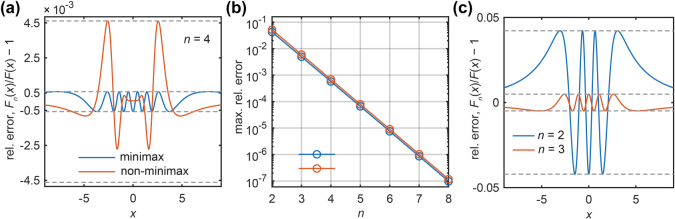
Minimax approximation error. (a) Error distribution of a *minimax rational approximation* and a non-minimax technique (Padé, [92]) for the Dawson function *F*(*x*), both using four poles (*n* = 4). The minimax approximation exhibits a characteristic equioscillating error – uniformly spread with alternating sign and constant magnitude and achieving optimal accuracy across the domain. The non-minimax (Padé) method shows lower error near the origin and at infinity but significantly higher (9× larger) global maximum error than the minimax. (b) Exponential convergence of the maximum relative error with increasing number of poles (*n*) for both the Dawson function and the Hilbert-reconstructed Faddeeva function approximations. With each additional pole, we observe an error reduction of roughly one order of magnitude. (c) Dawson’s approximation error for coefficients (23).

For example, for the Voigt distribution (18), the approximation coefficients [*B* ^*j*^, *C* ^*j*^] are calculated for the Faddeeva function *w*(*z*), with *n* being the number of approximation poles, Figure 3(c),19Notation: we use upper case for approximation constants (^*j*^); the lower index (_*i*_) is reserved for dispersion terms numbering, Eqs. (1–3, 5).

(23) $$\begin{array}{cc} \left(n=2\right)\;\;B^{1,2} & =0.28209\mp ı0.47633,\\ C^{1,2} & =-0.82576\pm ı0.57178;\\ \left(n=3\right)\;\;B^{1,2} & =-0.18872\mp 0.28646ı,\;B^{3}=0.94163,\\ C^{1,2} & =-1.00160\pm ı1.03731,\;C^{3}=-1.05117. \end{array}$$

Substituting the approximation (22) into the general susceptibility formula (5) gives a set of FDTD-compatible analytically derived dispersion terms

(24) $$\begin{array}{cc} \chi \left(t\right) & =a\varphi \left(t\right)\;\text{sin}\left(\Omega t-\phi \right)\theta \left(t\right)\\ & \approx \chi^{n}\left(t\right)=\overset{n}{\underset{j=1}{\sum }}a^{j}e^{-\gamma^{j}t}\sin\left(\Omega^{j}t-\phi^{j}\right)\theta \left(t\right)\;\;\overset{F}{\to }\\ \hat{\chi }\left(\omega \right) & =\frac{\iota \pi a}{2}\left[e^{ı\phi^{}}G\left(\omega ,\;,-,\;,\Omega \right)-e^{-ı\phi }\left(\omega ,\;,+,\;,\Omega \right)\right]\\ & \approx {\hat{\chi }}^{n}\left(\omega \right)=\overset{n}{\underset{j=1}{\sum }}\frac{a^{j}}{2}\left[\frac{e^{-ı\phi^{j}}}{\omega +ı\gamma^{j}+\Omega^{j}}-\frac{e^{ı\phi^{j}}}{\omega +ı\gamma^{j}-\Omega^{j}}\right], \end{array}$$

where parameters [*a* ^*j*^, *ϕ* ^*j*^, Ω^*j*^, *γ* ^*j*^] are directly connected to the parameters of the exact single-term model [*a*, *ϕ*, Ω, *γ*, *σ*] (5) and the approximation constants [*B*_*j*_, *C*_*j*_] in (22) as

(25) $$\left\{\begin{array}{cc} a^{j} & =a\sqrt{\pi }|B^{j}|,\\ \phi^{j} & =\phi -\text{Arg}\left(B^{j}\right),\\ \gamma^{j} & =\gamma -R\left[C^{j}\right]\sigma \sqrt{2},\\ \Omega^{j} & =\Omega +I\left[C^{j}\right]\sigma \sqrt{2}. \end{array}\right.$$

The MiMOSA model (24)–(25) retains the *single-oscillator form* [78], with only its envelope modified by approximation (compare to the exact susceptibility *χ*(*t*)),

(26) $$\chi^{n}\left(t\right)=a\underset{\approx \varphi \left(t\right)}{\underset{︸}{e^{-\gamma t}\left(\sqrt{\pi }R\overset{n}{\underset{j=1}{\sum }}B^{j}e^{C^{j}\sigma \sqrt{2}t}\right)}}\sin\left(\Omega t-\phi \right)\theta \left(t\right).$$

This identity arises by substituting coefficients (25) into the time-domain expression (24) and combining the conjugate pole pairs. It ensures that the model remains physically consistent, without introducing nonphysical oscillations.

Due to the *equioscillation theorem* [79], the minimax solution provides the shortest rational polynomial approximation (corresponding to most compact numerical stencil), with the approximation error spread evenly across the entire frequency domain. In the Voigt case $\sigma \sqrt{2\pi }G\left(z\sigma \sqrt{2};\gamma =0\right)=w\left(z\right)$ , the error converges exponentially with number of poles (*n*) throughout the upper half-plane [50]. As a result, even two poles (*n* = 2) already give a few percent error, which is sufficient for many applications, such as initial optimization or ellipsometry characterization. Using three poles (*n* = 3) drives the FD relative error below 1 %, making the approximation indiscernible from the experimental data (see Appendix C for details).

The MiMOSA models (24) fit the class of rational functions that can be coupled efficiently to the TD Maxwell’s solvers. Detailed ADE and RC numerical schemes for this class of dispersion can be found in Refs. [50], [80], [81] for second-order accurate TD solvers, and in Refs. [82], [83], [84] for higher-order schemes. We recommend using the *universal compact scheme*, which minimizes computational cost per dispersion term and enables easy switching between different second-order accurate ADE and RC formulations. An FDTD code implementing six such schemes is available in Ref. [50].

Compared to the approximations derived in the 1950s by reincarnating the minimax methods for rational polynomials and the more recent literature on the rational approximations to Faddeeva/Kramp/plasma dispersion function (or their real/imaginary parts) [85], [86], [87], [88], [89], [90], [91], [92], [93], [94], [95], [96], [97], [98], [99], [100], [101], [102], our MiMOSA method achieves impressive < 1 % error with just 2–3 terms (and thus the minimal number of additional equations in the numerical model), while preserving the necessary analytical properties of the dielectric function including causality and the sum rules.

A related computational approach has been recently proposed in Ref. [103], where an *ab initio* integral dispersion formulation is presented and subsequently transformed into a rational function through a quadrature approximation, thereby preserving the physical meaning of the main model parameters. However, no alternative approximation technique achieves the same minimal number of additional equations as MiMOSA for a given maximal error across the entire upper half-space.

## 4 Conclusions

This work advances the field of computational nanophotonics by introducing a general theoretical framework to model inhomogeneous broadening in disordered, defect-containing, and amorphous materials based on the absorption probability density functions *G*(*x*). The new formulation employs a complex absorption probability density, G=G(x)+ıH{G(x)} , in the frequency domain and a matching characteristic function $\varphi \left(t\right)=\int_{R}G\left(x\right)e^{ıxt}\;dx$ in the time domain.

Application examples of the theory include Gauss–Lorentz oscillator, Gauss–Debye relaxation, and Gauss–Drude conductivity models for the characterization and predictive modeling of inhomogeneous broadening effects in linear and nonlinear regimes and provide a critical fix to the noncausal Brendel–Bormann model (see Appendix B for comparison). The complete set of newly derived dispersion models is presented in Table A, Appendix A.

The exact generalized permittivity formulation is then used to obtain the efficient, best-possible minimax-based approximation (MiMOSA) models that enable (1) integral- and special-function-free permittivity calculation; (2) efficient FDTD implementation with a minimal set of the additional equations; and (3) ellipsometry fitting and lineshape retrieval. The MiMOSA implementation ensures efficient simulation while maintaining the desired controlled accuracy and analytical constraints.

The near-term work includes extending our approach to nonsymmetric distributions (e.g., the Fermi–Dirac distribution), and next-order corrections in the scattering function *γ*(*t*) = *γ* + *σ* ^2^ *t*/2 + ···, as well as developing the time-domain approximations to the widely used empirical non-symmetric models, including the Tauc(Cody)–Lorentz dispersion [27], [28] (Figure 2(a) and (b)). The proposed formulation for arbitrary probability density functions can become a foundational model for inhomogeneous broadening analysis. Its ability to retrieve broadening information through minimax coefficients and fitting to experimental data can provide invaluable insights into the lifetime-based width, local environments, and the nature of disorder in materials, thus improving our understanding of their fundamental properties [104].

Our approach extends naturally to anisotropic and bi-anisotropic materials involving full electromagnetic tensors, as well as to nonlinear models such as saturable Lorentz and multilevel carrier kinetics solvers, where the non-Lorentzian lineshapes can now be accurately implemented in FETD, DGTD, FVTD or FDTD solvers. Although this result focuses primarily on optical materials and nanophotonics, its implications extend broadly to wave propagation across various disciplines, including microwave electromagnetics, acoustics, electronics, magnonics, biosensing, seismology, astrophysics, and quantum information technologies, where our newly developed MiMOSA method efficiently accounts for inhomogeneous broadening in dispersive media.

## Appendix A: Table of susceptibility models with arbitrary broadening

Table A presents the *Fundamental Dispersion Model* (shown in the dark green cell) for any rational unbroadened dispersion function *χ* ^0^(.) broadened by an arbitrary Probability Density Function (PDF) *G*(*x*). Special cases of *χ* ^0^(.) are listed in the columns: *Oscillator* – difference of two complex conjugate or real poles (column 3), *Relaxation* – one real pole (column 4), *Conductivity* – difference of two real poles, one of which is zero (columns 5). Special cases of the broadening function *G*(*x*) are listed in the rows: the most general case is *Any Broadening* (row 2) with given Characteristic Function (CF) $\varphi \left(t\right)=\int_{-\infty }^{\infty }G\left(x\right)e^{ıxt}dx$ and complex PDF G(x)=G(x)+ıH{G(x)} , followed by the *Voigt profile* (row 3), and its subcases – pure Gauss and pure Lorentz (Cauchy) broadening (rows 4–5), then the unbroadened case *G*(*x*) = *δ*(*x*) (row 6). In the time domain, broadening means multiplication of the unbroadened susceptibility *χ* ^0^(*t*) by the CF *φ*(*t*). In the frequency domain, this translates to the convolution of ${\hat{\chi }}^{0}\left(\omega \right)$ with the PDF *G*(*x*), which can be expressed in terms of the complex PDF G(x) . Each cell represents a special case of the *fundamental model* (dark green cell), obtained by substitution of [*a*, *ϕ*, Ω, *γ*, *σ*]-parameters (shown in the header) and [φ(t),G(x)] -functions (shown in column 1). The cell color legend is as follows: light red indicates agreement with long-established classical models, while greenish cells denote formulations newly introduced in this work. The medium green cell corresponds to a recent result (obtained both here and in Ref. [74]) that provides an important correction to the well-known noncausal Brendel–Bormann model (see Appendix B for details).

**Table:**

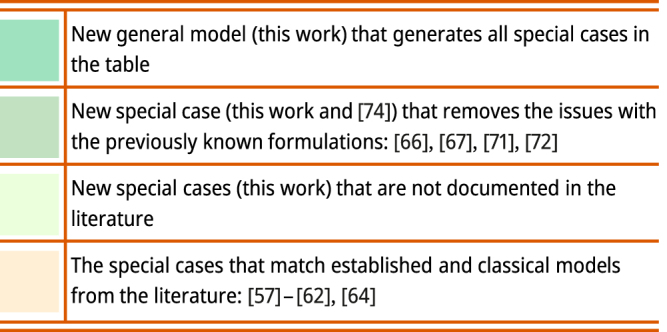

**Table A:** Susceptibility $\hat{\chi }\left(\omega \right)=\left({\hat{\chi }}^{0}*G\right)\left(\omega \right)$ for different unbroadened dispersion ${\hat{\chi }}^{0}\left(\omega \right)$ (columns) and broadening functions G(x)=R[G(x)] (rows). For conductivity, $\hat{\sigma }\left(\omega \right)=\left({\hat{\sigma }}^{0}*G\right)\left(\omega \right)$ , then restore $\chi \left(t\right)=\varepsilon_{0}^{-1}\int_{0}^{t}\sigma \left(\tau \right)d\tau$ .

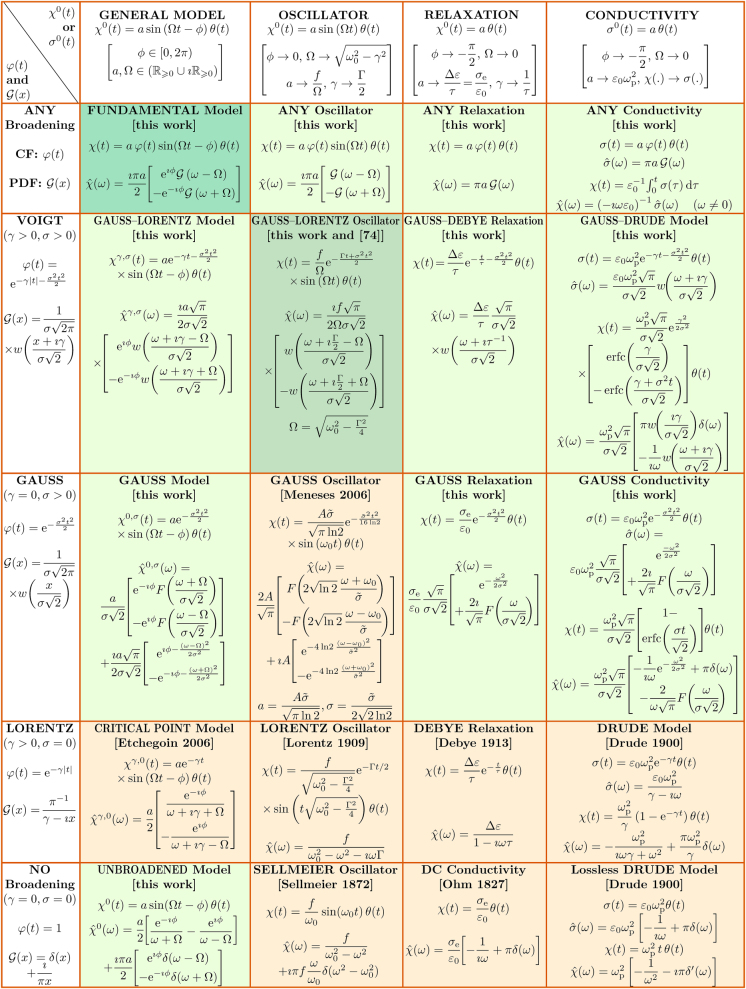

**The general model** is parameterized by the *time-domain* phase *ϕ*, amplitude *a*, resonance frequency Ω, and broadening (*σ*, *γ*) parameters, yielding simple formulas. The phase parameter *ϕ* allows to account for a critical point (CP) model [60], and toggles between two orthogonal cases: a relaxation (*ϕ* = −*π*/2) with one real pole *s*_1_ = −*γ*, and an oscillator (*ϕ* = 0) with two poles *s*_1,2_ = −*γ* ± *ı*Ω, where $\Omega =\sqrt{\omega_{0}^{2}-\gamma^{2}}$ , and *s* = −*ıω* is polynomial argument. Two poles are either complex conjugates (*γ* < *ω*_0_) or both real (*γ* ≥ *ω*_0_); the latter is called an *overdamped oscillator*. If one real pole is zero (*ω*_0_ = 0), the model represents conductivity case. To correctly take the limits *γ* → 0^+^ and *σ* → 0^+^ in the general model columns, use the following tips:

(A.1a)
$$\gamma \to 0^{+}:\text{ for }x\in R,\text{the real and imaginary parts separate as }w\left(x\right)=e^{-x^{2}}+\frac{2ı}{\sqrt{\pi }}F\left(x\right).$$

(A.1b)
$$\sigma \to 0^{+}:\text{ for large argument use }w\left(z\right)\approx \frac{ı}{z\sqrt{\pi }}\text{ and }\text{erfc}\left(z\right)=e^{-z^{2}}w\left(ız\right)\approx \frac{e^{-z^{2}}}{z\sqrt{\pi }}.$$

(A.1c)
$$\gamma \to 0^{+},\sigma \to 0^{+}:\text{ use the Sokhotski}-\text{Plemelj theorem to obtain Zero Broadening formulas.}$$

**Oscillator**  (*ϕ* = 0, Ω > 0) is usually defined by the natural frequency *ω*_0_, broadening Γ, and oscillator strength *f*, as in $\hat{\chi }\left(\omega \right)=\frac{f}{\omega_{0}^{2}-\omega^{2}-ı\omega \Gamma }$ . In the time domain, it represents either an oscillator (if the resonance frequency is real, $\Omega =\sqrt{\omega_{0}^{2}-\Gamma^{2}/4}\in R$ ), or a difference of two exponential decays (overdamped oscillator), otherwise (Γ/2 > *ω*_0_).

**Relaxation** (*ϕ* = −*π*/2, Ω = 0) corresponds to exponential decay in the time domain. Its amplitude is classically characterized by a permittivity jump Δ*ɛ* at *ω* = 0, with the fall rate defined by the relaxation time *τ*, as in $\hat{\chi }\left(\omega \right)=\frac{\Delta \varepsilon }{1-ı\omega \tau }$ . In the zero broadening limit *γ* = *τ* ^−1^ → 0^+^, this parametrization becomes ill-defined, so a DC electric conductivity parameter is used instead, *σ*_e_ = *ɛ*_0_Δ*ɛτ* ^−1^, for the Gaussian and zero broadening cases.

**Conductivity** (*ϕ* = −*π*/2, Ω = 0, *χ*(.) → *σ*(.)) is characterized by the plasma frequency *ω*_p_ and collision rate *γ*, as in $\hat{\chi }\left(\omega \right)=-\frac{\omega_{\text{p}}^{2}}{\omega^{2}+ı\gamma \omega }=\frac{\omega_{\text{p}}^{2}}{\gamma }\left[\frac{1}{-ı\omega }-\frac{1}{\gamma -ı\omega }\right]$ , (*ω* ≠ 0). An alternative (equivalent) definition using the conductivity function is $\hat{\sigma }\left(\omega \right)=-ı\omega \varepsilon_{0}\hat{\chi }\left(\omega \right)=\frac{\varepsilon_{0}\omega_{\text{p}}^{2}}{\gamma -ı\omega }$ . In conductive media, broadening is applied to the conductivity function $\hat{\sigma }\left(\omega \right)$ rather than to the susceptibility $\hat{\chi }\left(\omega \right)$ , as is done in non-conductive cases (this ensures that the zero pole remains unbroadened); thus the substitution *χ*(.) → *σ*(.) is used.

## Appendix B: Correction to the Brendel–Bormann (BB) model

Efimov and Khitrov [66] and later Brendel and Bormann [67] postulated that the following convolution integral introduces the Voigt (Gauss–Lorentz) broadening to the classical Lorentz oscillator,

(B.2)
$$\begin{array}{ll} {\hat{\chi }}_{\text{BB}}\left(\omega \right) & =\frac{1}{\sigma \sqrt{2\pi }}\overset{+\infty }{\underset{-\infty }{\int }}\frac{f}{x^{2}-\omega^{2}-ı\omega \Gamma }\\ & \;\times \exp\left(-\frac{{\left(x-\omega_{0}\right)}^{2}}{2\sigma^{2}}\right)\;dx. \end{array}$$

This integral can be solved in terms of Faddeeva functions, as shown in Rakić et al. [68],

(B.3)
$$\begin{array}{ll} {\hat{\chi }}_{\text{BB}}\left(\omega \right) & =\frac{ı\;f\sqrt{\pi }}{\sigma \sqrt{2}}\;\frac{1}{2a\left(\omega \right)}\left[w\left(\frac{a\left(\omega \right)-\omega_{0}}{\sigma \sqrt{2}}\right)\right.\\ & \;\left.+w\left(\frac{a\left(\omega \right)+\omega_{0}}{\sigma \sqrt{2}}\right)\right],\\ a\left(\omega \right) & =\sqrt{\omega^{2}+ı\omega \Gamma },\;\left(Ia\left(\omega \right)\geq 0\right). \end{array}$$

While the BB model (B.2) and (B.3) can be useful in specific cases of experimental frequency-domain spectroscopy, e.g., [68], it is inherently non-causal. This drawback restricts its utility primarily to spectral fitting applications and makes it unsuitable for time-domain simulations. The properties of the BB model and possible corrections have been discussed in the literature up to today, [71], [72], [73].

In this work, we have built a physically consistent formalism for susceptibility functions broadened by any absorption probability *G*(*x*), including Voigt profile. First, we express the Lorentz oscillator with strength *f*, natural frequency *ω*_0_, and (homogeneous) broadening *γ* = Γ/2 in the time and frequency domains,

(B.4)
$$\begin{array}{ll} \chi_{\text{L}}\left(t\right) & =\frac{f}{\Omega }e^{-\gamma t}\sin\left(\Omega t\right)\theta \left(t\right)\;\;\;\;\overset{F}{\to }\\ {\hat{\chi }}_{\text{L}}\left(\omega \right) & =\frac{f}{\omega_{0}^{2}-\omega^{2}-2ı\omega \gamma },\;\;\;\left(\Omega =\sqrt{\omega_{0}^{2}-\gamma^{2}}\right). \end{array}$$

Second, we write the Gaussian *probability density function* (PDF) and corresponding *characteristic function* (CF), both characterized by the variance *σ* ^2^,

(B.5a)
$$PDF:\;\;\;G\left(x;\mu =0,\sigma^{2}\right)=\frac{1}{\sigma \sqrt{2\pi }}e^{-\frac{x^{2}}{2\sigma^{2}}},$$

(B.5b)
$$CF:\;\;\;\varphi \left(t\right)=\overset{+\infty }{\underset{-\infty }{\int }}G\left(x\right)e^{ıxt}dx=e^{-\frac{\sigma^{2}}{2}t^{2}}.$$

Note that we assume zero mean (*μ* = 0) which keeps the resonance frequency Ω of the oscillator unshifted.

In the time domain, the Gauss–Lorentz model is a multiplication of the Lorentz oscillator (B.4) by the Gaussian CF (B.5b),

(B.6)
$$\begin{array}{ll} \chi_{\text{GL}}\left(t\right) & =\underset{\text{Lorentz}}{\underset{︸}{\frac{f}{\Omega }e^{-\gamma t}\sin\left(\Omega t\right)\theta \left(t\right)}}\underset{\text{Gaussian CF}}{\underset{︸}{e^{-\frac{\sigma^{2}}{2}t^{2}}}}\\ & =\underset{\text{Sellmeier}}{\underset{︸}{\frac{f}{\Omega }\sin\left(\Omega t\right)\theta \left(t\right)}}\underset{\text{Cauchy CF}}{\underset{︸}{e^{-\gamma t}}}\underset{\text{Gaussian CF}}{\underset{︸}{e^{-\frac{\sigma^{2}}{2}t^{2}}}}, \end{array}$$

which can also be viewed as a lossless Lorentz (Sellmeier) oscillator broadened by both Cauchy and Gaussian distributions. This aligns with the general principle from probability theory: the CF of the sum of two random variables is a product of individual CFs, while the PDF of the sum is a convolution. Equation (B.6) preserves causality (note the term *θ*(*t*)) and leads to an *inhomogeneous* time-dependent scattering function *γ*(*t*) = *γ* + *σ* ^2^ *t*/2, where higher order correction terms are possible for other broadening functions *G*(*x*), as predicted by Kim et al. [24].

In the frequency domain, according to the convolution theorem, such multiplication corresponds to the integral

(B.7)
$$\begin{array}{ll} {\hat{\chi }}_{\text{GL}}\left(\omega \right) & =\left({\hat{\chi }}_{\text{L}}*G\right)\left(\omega \right)\\ & =\overset{+\infty }{\underset{-\infty }{\int }}\underset{\text{Lorentz }{\hat{\chi }}_{\text{L}}\left(\omega -x\right)}{\underset{︸}{\frac{f}{\omega_{0}^{2}-{\left(\omega -x\right)}^{2}-2ı\left(\omega -x\right)\gamma }}}\underset{\text{Gauss PDF G}\left(x;0,\sigma^{2}\right)}{\underset{︸}{\frac{1}{\sigma \sqrt{2\pi }}e^{-\frac{x^{2}}{2\sigma^{2}}}}}\;\;dx. \end{array}$$

Decomposing the Lorentzian into single poles (with $\omega_{0}^{2}=\Omega^{2}+\gamma^{2}$ ) gives

$$\begin{array}{ll} \frac{f}{\omega_{0}^{2}-{\left(\omega -x\right)}^{2}-2ı\left(\omega -x\right)\gamma } & =\frac{f}{2\Omega }\left[\frac{1}{\omega +ı\gamma -x+\Omega }\right.\\ & \;\left.-\frac{1}{\omega +ı\gamma -x-\Omega }\right], \end{array}$$

making substitution *x* → −*x* in the integral for the first pole, and then (*x* + Ω) → *x* for both poles, gives the final integral (where Γ = 2*γ*)

(B.8)
$$\begin{array}{ll} {\hat{\chi }}_{\text{GL}}\left(\omega \right)= & \frac{1}{\sigma \sqrt{2\pi }}\overset{+\infty }{\underset{-\infty }{\int }}\frac{x}{\Omega }\;\;\frac{f}{x^{2}-\omega^{2}-ı\omega \Gamma +\Gamma^{2}/4}\\ & \;\times \exp\left(-\frac{{\left(x-\Omega \right)}^{2}}{2\sigma^{2}}\right)dx, \end{array}$$

and its closed-form expression in terms of the Faddeeva functions

(B.9)
$$\begin{array}{ll} {\hat{\chi }}_{\text{GL}}\left(\omega \right) & =\frac{ı\;f\sqrt{\pi }}{\sigma \sqrt{2}}\;\frac{1}{2\Omega }\left[w\left(\frac{\omega +ı\Gamma /2-\Omega }{\sigma \sqrt{2}}\right)\right.\\ & \;\left.-w\left(\frac{\omega +ı\Gamma /2+\Omega }{\sigma \sqrt{2}}\right)\right], \end{array}$$

(B.10)
$$\begin{array}{ll} {\hat{\chi }}_{\text{BB}}\left(\omega \right) & =\frac{ı\;f\sqrt{\pi }}{\sigma \sqrt{2}}\;\frac{1}{2a\left(\omega \right)}\left[w\left(\frac{a\left(\omega \right)-\omega_{0}}{\sigma \sqrt{2}}\right)\right.\\ & \;\left.+w\left(\frac{a\left(\omega \right)+\omega_{0}}{\sigma \sqrt{2}}\right)\right],\;\;\;a\left(\omega \right)=\sqrt{\omega^{2}+ı\omega \Gamma }. \end{array}$$

Comparison of the GL model (B.9) with the BB model (B.3) (duplicated in (B.10) for convenience) indicates two key differences:

1. The resonance and natural frequencies are confused, Ω ≠ *ω*_0_. For a mildly damped oscillator Γ ≪ *ω*_0_ this can be a close approximation, $\Omega =\sqrt{\omega_{0}^{2}-\Gamma^{2}/4}\approx \omega_{0}$ . Similarly, for high enough frequencies *ω* ≫ Γ, we have $a\left(\omega \right)=\sqrt{{\left(\omega +ı\Gamma /2\right)}^{2}+\Gamma^{2}/4}\approx \omega +ı\Gamma /2$ .
2. The BB model uses a sum of the Faddeeva functions instead of a difference. For the Lorentzians, the sum and difference are identical,
  $$\frac{1}{a}\left[\frac{1}{a-\Omega }+\frac{1}{a+\Omega }\right]=\frac{1}{\Omega }\left[\frac{1}{a-\Omega }-\frac{1}{a+\Omega }\right].$$
  For the Voigt profile, same identity does not hold, i.e.,
  $$\frac{1}{a}\left[w\left(a-\Omega \right)+w\left(a+\Omega \right)\right]\neq \frac{1}{\Omega }\left[w\left(a-\Omega \right)-w\left(a+\Omega \right)\right].$$
  Only with a negligible Gaussian width, *σ* ≪ Γ, the Voigt profile simplifies to a Lorentzian, and the sum can approximate the difference, which can be shown using the asymptotic formula of large arguments, *w*(*z*) ≈ *ıπ* ^−1/2^ *z* ^−1^. As a result, the BB model (B.3) can be useful for Gaussian broadening analysis but only becomes close to the true GL formula for feebly damped (Γ ≪ *ω*_0_) and feebly Gaussian (*σ* ≪ Γ) oscillators over higher frequency ranges (*ω* ≫ Γ). The BB model (B.3) violates causality, which makes it unusable in time-domain simulations. Instead, the Gauss–Lorentz (GL) model (B.8) and (B.9) should be used for spectral analysis and simulations, especially in the time domain.

The new GL model is causal (*χ*_GL_(*t*) = 0 ∀*t* < 0), has the correct symmetry ${\hat{\chi }}_{\text{GL}}\left(-\omega \right)={\hat{\chi }}_{\text{GL}}^{*}\left(\omega \right)$ , preserves the Lorentz plasma sum rule: ${\hat{\chi }}_{\text{GL}}\left(\omega \right)\approx -f/\omega^{2}\;\text{ as }\omega \to \infty$ , and has correct pure Lorentz (*σ* → 0^+^) and pure Gaussian (*γ* → 0^+^) limits, as follows from properties of the Faddeeva function (*w*(−*z*) = *w**(*z**) and *w*(*z*) ≈ *ıπ* ^−1/2^ *z* ^−1^).

## Appendix C: Equioscillation theorem and MiMOSA method

The minimax optimization technique utilized in the MiMOSA method traces its historical origins to the 19th century work of Pafnuty Tchebycheff (Chebyshev) [79]. The *equioscillation theorem*, also known as the Chebyshev alternation theorem, represents a fundamental principle in approximation theory. It states that, when approximating a continuous function, the optimal uniform (minimax) rational approximation of degree [*M*, *N*] exhibits a distinctive pattern: the approximation error attains its maximum absolute value at least *M* + *N* + 2 times across the interval. At these extremal points, the error alternates precisely in sign and has equal magnitude, hence the term “equioscillation”. This evenly distributed alternation of maximal error is the defining feature of the optimal solution in the minimax sense, see Figure 3(a).

The MiMOSA method starts by finding such optimal *minimax rational approximation* for the Hilbert transform of the probability function H{G(x)} along the real axis, with a *sum rule constraint* imposed at infinity. This choice is motivated by the fact that while the absorption ${\hat{\chi }}^{\prime \prime }\left(\omega \right)$ may lack a rational asymptote at infinity (e.g., Gaussian absorption decays as $e^{-\omega^{2}/2\sigma^{2}}$ ), its Hilbert transform decays as ${\hat{\chi }}^{\prime }\left(\omega \right)∼-\frac{\omega_{\text{p}}^{2}}{\omega^{2}}$ according to the sum rule (Section 2.2).

Figure 3(a) illustrates the minimax concept and its advantages over non-minimax approximation methods. Shown is a relative error of the sum-rule-constrained 4-pole (*n* = 4) minimax rational approximation of the Dawson function (*F*(*x*) ≈ *F*_*n*_(*x*)), featuring (4*n* − 1) equioscillating peaks (in agreement with the alternation theorem). As a non-minimax reference, we include a 4-pole Padé approximation [92], a method that has recently gained popularity in computational modeling [100]. While the Padé approximation achieves higher precision near *x* = 0 and at infinity, its global maximum error in this example is 9 times larger (4.5e-3) than that of the minimax approximation (0.5e-3). In time-domain simulations, particularly those involving ultrafast phenomena, broadband accuracy is essential, making the minimax approach optimal for achieving the best overall accuracy with a fixed number of poles *n*.

Figure 3(b) demonstrates that the maximum relative error of the Dawson function approximation, $\underset{x}{\max}|F_{n}\left(x\right)/F\left(x\right)-1|$ decreases exponentially with the number of poles *n*. Furthermore, reconstructing the real part via the Hilbert transform,

$$w_{n}\left(z\right)=\frac{2}{\sqrt{\pi }}\left(ıF_{n}\left(z\right)+H^{-1}\left\{F_{n}\left(z\right)\right\}\right),$$

yields an approximation of the Faddeeva function *w*(*z*) with approximately the same maximum relative error across the *entire upper half-plane*,

$$\underset{z\in C,I\left[z\right]\geq 0}{\max}\left|\frac{w_{n}\left(z\right)}{w\left(z\right)},-,1\right|.$$

Each additional pole reduces the error by roughly *an order of magnitude*, highlighting the rapid convergence of the minimax approximation.

Described properties are essential to the efficiency of MiMOSA permittivity models for the following reasons:

– **Optimal error distribution.** MiMOSA models use minimax rational approximations to uniformly minimize error across the spectral domain, ensuring consistent broadband accuracy without localized degradation.
– **Minimal number of poles.** High accuracy (better than 1 %) can be achieved with just 2–3 poles, significantly reducing computational cost. A smaller number of poles translates into the shortest possible time-domain stencil in the FDTD update equations, which is critical for fast and memory-efficient time-domain simulations.
– **Physically consistent formulation.** The semi-analytical derivation with built-in constraints (e.g., causality, sum rules, Kramers–Kronig consistency) ensures that MiMOSA models maintain the structure and interpretability of *a single oscillator.* Unlike overfitted multi-parameter models, the compact form of MiMOSA improves fitting stability and gives physical meaning to each parameter.

## Appendix D: Abbreviations and functions

PDF – Probability density function, *G*(*x*); it is nonnegative (*G*(*x*) ≥ 0) and has full probability support $\left(\int_{R},G,(,x,),\text{d},x,=,1\right)$ . *Examples*: Cauchy/Lorentz $G_{L}\left(x\right)=\frac{1}{\pi }\frac{\gamma }{{\left(x-\mu \right)}^{2}+\gamma^{2}}$ and Gauss $G_{G}\left(x\right)=\frac{1}{\sigma \sqrt{2\pi }}e^{-\frac{{\left(x-\mu \right)}^{2}}{2\sigma^{2}}}$ .

Complex PDF – Probability density function with added Hilbert transform as an imaginary part, G(x)=G(x)+ıH{G(x)} . *Examples* (*μ* = 0): Cauchy/Lorentz $G_{\text{L}}\left(x\right)=\frac{\pi^{-1}}{\gamma -ıx}$ and Gauss $G_{\text{G}}\left(x\right)=\frac{1}{\sigma \sqrt{2\pi }}w\left(\frac{x}{\sigma \sqrt{2}}\right)$ .

CF – Characteristic function, $\varphi \left(t\right)=\overset{\infty }{\underset{-\infty }{\int }}G\left(x\right)e^{ıxt}dx$ ; it is bounded $\left(|\varphi \left(t\right)|\leq 1\right)$ and zero-centered $\left(\varphi \left(0\right)=1\right)$ ; moreover, if the PDF is symmetric, its CF is real-valued. *Examples* (*μ* = 0): Cauchy/Lorentz *φ*_L_(*t*) = e^−*γ*|*t*|^ and Gauss $\varphi_{\text{G}}\left(t\right)={\text{e}}^{-\sigma^{2}t^{2}/2}$ .

sPDF – standard PDF, *g*(*x*) = *G*(*x*;*μ*=0,*σ* ^2^=1) – a normalized PDF, with the argument centered and stretched such that: the mean is zero (*μ* = 0) and variance is one (*σ* = 1) leading to $G\left(x;\mu ,\sigma^{2}\right)=\frac{1}{\sigma }g\left(\frac{x-\mu }{\sigma }\right)$ . *Examples*: Cauchy/Lorentz $g_{\text{L}}\left(x\right)=\frac{\pi^{-1}}{x^{2}+1}$ and Gauss $g_{\text{G}}\left(x\right)=\frac{1}{\sqrt{2\pi }}{\text{e}}^{-x^{2}/2}$ .

Lineshape, *l*(*x*) – a normalized distribution with the argument centered and stretched and the amplitude scaled so that: the peak is centered at zero with maximum of 1 and half-width-half-maximum (HWHM) of 1. *Examples*: Cauchy/Lorentz $l_{\text{L}}\left(x\right)=\frac{1}{1+x^{2}}$ and Gauss $l_{\text{G}}\left(x\right)={\text{e}}^{-x^{2}\ln2}$ .

CP – Critical point model, known in the semiconductor literature [60].

FT – Fourier transform (F) , $F\left\{f\left(t\right)\right\}=\hat{f}\left(\omega \right)=\overset{\infty }{\underset{-\infty }{\int }}f\left(t\right)e^{ı\omega t}\;\text{d}t$ .

IFT – Inverse Fourier transform $\left(F^{-1}\right)$ , $F^{-1}\left\{\hat{f}\left(\omega \right)\right\}=f\left(t\right)={\left(2\pi \right)}^{-1}\overset{\infty }{\underset{-\infty }{\int }}\hat{f}\left(\omega \right)e^{-ı\omega t}\;d\omega$ .

HT – Hilbert transform (H) , $H\left\{f\left(x\right)\right\}=\pi^{-1}P\overset{\infty }{\underset{-\infty }{\int }}\frac{f\left(t\right)}{x-t}\;\text{d}t$ .

IHT – Inverse Hilbert transform $\left(H^{-1}\right)H^{-1}\left\{f\left(x\right)\right\}=-H\left\{f\left(x\right)\right\}$ .

TD – Time domain.

FD – Frequency domain.

ZB – Zero broadening (*γ* = 0^+^, *σ* = 0^+^).

HB – Homogeneous broadening (*γ* > 0, *σ* = 0^+^).

IB – Inhomogeneous broadening (*γ* ≥ 0, *σ* > 0).

The Faddeeva (or Kramp) function, $w\left(z\right)=e^{-z^{2}}\text{erfc}\left(-ız\right)=e^{-z^{2}}+\frac{2ı}{\sqrt{\pi }}F\left(z\right)$ , [63].

The Dawson function (or Dawson integral), $F\left(x\right)=\int_{0}^{x}e^{t^{2}-x^{2}}\;dt=H\left\{\frac{\sqrt{\pi }}{2}e^{-x^{2}}\right\}=\frac{\sqrt{\pi }}{2}I\left[w\left(x\right)\right]$ , *x* ∈ R .

Conductivity function $\hat{\sigma }\left(\omega \right)=-ı\omega \varepsilon_{0}\hat{\chi }\left(\omega \right)$ , *σ*(*t*) = *ɛ*_0_ *χ*′(*t*), $\chi \left(t\right)=\varepsilon_{0}^{-1}\int_{0}^{t}\sigma \left(\tau \right)d\tau$ .

The Dirac delta function, *δ*(*x*).

The Heaviside step function, *θ*(*t*).

## References

[1] Demtroder W. (2003). *Laser Spectroscopy: Basic Concepts and Instrumentation*.

[2] Weisskopf V., Wigner E. (1930). Uber die naturliche linienbreite in der strahlung des harmonischen oszillators. *Z. Phys.*.

[3] Spinelli P., Verschuuren M. A., Polman A. (2012). Broadband omnidirectional antireflection coating based on subwavelength surface Mie resonators. *Nat. Commun.*.

[4] Chowdhury S. N. (2024). Wide-range angle-sensitive plasmonic color printing on lossy-resonator substrates. *Adv. Optical Mater.*.

[5] Simon J., Fruhling C., Kim H., Gogotsi Y., Boltasseva A. (2023). MXenes for optics and photonics. *Opt. Photonics News*.

[6] Narayanan M., Shah A. P., Ghosh S., Thamizhavel A., Bhattacharya A. (2023). Elucidating the role of oxygen vacancies on the electrical conductivity of *β*-Ga_2_O_3_ single-crystals. *Appl. Phys. Lett.*.

[7] Anderson P. W. (1952). A method of synthesis of the statistical and impact theories of pressure broadening. *Phys. Rev.*.

[8] Mukamel S. (1995). *Principles of Nonlinear Optical Spectroscopy*.

[9] Stoneham A. M. (1975). *Theory of Defects in Solids*.

[10] Bimberg D., Grundmann M., Ledentsov N. N. (1999). *Quantum Dot Heterostructures*.

[11] Elliott S. R. (1990). *Physics of Amorphous Materials*.

[12] Dicke R. H. (1953). The effect of collisions upon the Doppler width of spectral lines. *Phys. Rev.*.

[13] Piccardo M. (2020). Frequency combs induced by phase turbulence. *Nature*.

[14] Demangeot F., Simeonov D., Dussaigne A., Butté R., Grandjean N. (2009). Homogeneous and inhomogeneous linewidth broadening of single polar GaN/AlN quantum dots. *Phys. Status Solidi C*.

[15] Faist J. (1993). Measurement of the intersubband scattering rate in semiconductor quantum wells by excited state differential absorption spectroscopy. *Appl. Phys. Lett.*.

[16] Capasso F., Faist J., Sirtori C. (1996). Mesoscopic phenomena in semiconductor nanostructures by quantum design. *J. Math. Phys.*.

[17] Lifshitz I. M. (1964). The energy spectrum of disordered systems. *Adv. Phys.*.

[18] John S. (1987). Strong localization of photons in certain disordered dielectric superlattices. *Phys. Rev. Lett.*.

[19] Juve V. (2013). Size-dependent surface plasmon resonance broadening in nonspherical nanoparticles: single gold nanorods. *Nano Lett.*.

[20] Foteinopoulou S., Devarapu G. C. R., Subramania G. S., Krishna S., Wasserman D. (2019). Phonon-polaritonics: enabling powerful capabilities for infrared photonics. *Nanophotonics*.

[21] Fujiwara H. (2007). *Spectroscopic Ellipsometry: Principles and Applications*.

[22] Woollam J. A. (2020). *CompleteEASE Software Manual*.

[23] Thompson P., Cox D. E., Hastings J. B. (1987). Rietveld refinement of Debye–Scherrer synchrotron X-ray data from Al_2_O_3_. *J. Appl. Crystallogr.*.

[24] Kim C. C., Garland J. W., Abad H., Raccah P. M. (1992). Modeling the optical dielectric function of semiconductors: extension of the critical-point parabolic-band approximation. *Phys. Rev. B*.

[25] Kim C. C., Garland J. W., Raccah P. M. (1993). Modeling the optical dielectric function of the alloy system Al_x_Ga_x_As. *Phys. Rev. B*.

[26] Rakic A. D., Majewski M. L. (1996). Modeling the optical dielectric function of GaAs and AlAs: extension of Adachi’s model. *J. Appl. Phys.*.

[27] Jellison G. E., Modine F. A. (1996). Parameterization of the optical functions of amorphous materials in the interband region. *Appl. Phys. Lett.*.

[28] Ferlauto A. S. (2002). Analytical model for the optical functions of amorphous semiconductors from the near-infrared to ultraviolet: applications in thin film photovoltaics. *J. Appl. Phys.*.

[29] Tanguy C. (1995). Optical dispersion by Wannier excitons. *Phys. Rev. Lett.*.

[30] Tanguy C. (1999). Analytical expression of the complex dielectric function for the Hulthén potential. *Phys. Rev. B*.

[31] Krausz F., Ivanov M. (2009). Attosecond physics. *Rev. Mod. Phys.*.

[32] Pelton M. (2015). Modified spontaneous emission in nanophotonic structures. *Nat. Photonics*.

[33] Awschalom D. D., Hanson R., Wrachtrup J., Zhou B. B. (2018). Quantum technologies with optically interfaced solid-state spins. *Nat. Photonics*.

[34] Alcorn F. M., Jain P. K., van der Veen R. M. (2023). Time-resolved transmission electron microscopy for nanoscale chemical dynamics. *Nat. Rev. Chem.*.

[35] Moerner W. E., Orrit M. (1999). Illuminating single molecules in condensed matter. *Science*.

[36] Shah J. (1999). *Ultrafast Spectroscopy of Semiconductors and Semiconductor Nanostructures*.

[37] Jonas D. M. (2003). Two-dimensional femtosecond spectroscopy. *Annu. Rev. Phys. Chem.*.

[38] Wright J. C. (2010). Coherent multidimensional vibrational spectroscopy. *Int. Rev. Phys. Chem.*.

[39] Mastron J. N., Tokmakoff A. (2018). Fourier transform fluorescence-encoded infrared spectroscopy. *J. Phys. Chem. A*.

[40] Tinnefeld P., Eggeling C., Hell S. W. (2015). *Far-Field Optical Nanoscopy*.

[41] Luebbers R. J., Hunsberger F. P., Kunz K. S., Standler R. B., Schneider M. (1990). A frequency-dependent finite-difference time-domain formulation for dispersive materials. *IEEE Trans. Electromagn. Compat.*.

[42] Kashiwa T., Fukai I. (1990). A treatment by the FDTD method of the dispersive characteristics associated with electronic polarization. *Microw. Opt. Technol. Lett.*.

[43] Joseph R. M., Hagness S. C., Taflove A. (1991). Direct time integration of Maxwell’s equations in linear dispersive media with absorption for scattering and propagation of femtosecond electromagnetic pulses. *Opt. Lett.*.

[44] Young J. L. (1995). Propagation in linear dispersive media: finite difference time-domain methodologies. *IEEE Trans. Antennas Propag.*.

[45] Bui M. D., Stuchly S. S., Costache G. I. (1991). Propagation of transients in dispersive dielectric media. *IEEE Trans. Microw. Theory Tech.*.

[46] Kelley D. F., Luebbers R. J. (1996). Piecewise linear recursive convolution for dispersive media using FDTD. *IEEE Trans. Antennas Propag.*.

[47] Schuster J. W., Luebbers R. J. (1998). An accurate FDTD algorithm for dispersive media using a piecewise constant recursive convolution technique. *Proc. IEEE AP-S International Symposium*.

[48] Siushansian R., LoVetri J. (1997). Efficient evaluation of convolution integrals arising in FDTD formulations of electromagnetic dispersive media. *J. Electromagn. Waves Appl.*.

[49] Sullivan D. M. (1992). Frequency-dependent FDTD methods using Z transforms. *IEEE Trans. Antennas Propag.*.

[50] Prokopeva L. J., Peana S., Kildishev A. V. (2022). Gaussian dispersion analysis in the time domain: efficient conversion with Padé approximants. *Comput. Phys. Commun.*.

[51] Franta D., Necas D., Zajickova L., Ohlidal I. (2014). Broadening of dielectric response and sum rule conservation. *Thin Solid Films*.

[52] Bruus H., Flensberg K. (2004). *Many-Body Quantum Theory in Condensed Matter Physics: An Introduction*.

[53] Bekefi G., Barrett A. H. (1977). *Electromagnetic Vibrations, Waves, and Radiation*.

[54] Durrett R. (2019). *Probability: Theory and Examples*.

[55] Smith D. Y., Palik E. D. (1997). Dispersion theory, sum rules, and their application to the analysis of optical data. *Handbook of Optical Constants of Solids*.

[56] Franta D., Vohanka J., Hroncova B. (2023). Dispersion models exhibiting natural optical activity: theory of the dielectric response of isotropic systems. *J. Opt. Soc. Am. B*.

[57] Sellmeier W. (1872). Ueber die durch die aetherschwingungen erregten mitschwingungen der koerpertheilchen und deren rueckwirkung auf die ersteren, besonders zur erklaerung der dispersion und ihrer anomalien. *Ann. Phys. (Berlin, Ger.)*.

[58] Ohm G. S. (1827). *Die Galvanische Kette, Mathematisch Bearbeitet*.

[59] Drude P. (1900). Zur elektronentheorie der metalle. *Ann. Phys. (Berlin, Ger.)*.

[60] Etchegoin P. G., Le Ru E. C., Meyer M. (2006). An analytic model for the optical properties of gold. *J. Chem. Phys.*.

[61] Lorentz H. A. (1909). *The Theory of Electrons and Its Applications to the Phenomena of Light and Radiant Heat*.

[62] Debye P. (1913). Zur theorie der anomalen dispersion im gebiete der langwelligen elektrischen strahlung. *Ber. Dtsch. Phys. Ges.*.

[63] Faddeyeva V. N., Terentev N. M. (1961). *Tables of Values of the Function 
$w\left(z\right)=e^{-z^{2}}\left(1+\frac{2i}{\sqrt{\pi }}\overset{z}{\underset{0}{\int }}e^{t^{2}}dt\right)$
 for Complex Argument*.

[64] De Sousa Meneses D., Malki M., Echegut P. (2006). Structure and lattice dynamics of binary lead silicate glasses investigated by infrared spectroscopy. *J. Non-Cryst. Solids*.

[65] Peiponen K.-E., Vartiainen E. M. (1991). Kramers-Kronig relations in optical data inversion. *Phys. Rev. B*.

[66] Efimov A., Khitrov V. (1979). Analytical formulas for describing the dispersion of glass with refractive indices that observe the continuous nature of absorption. *Fiz. Khim. Stekla*.

[67] Brendel R., Bormann D. (1992). An infrared dielectric function model for amorphous solids. *J. Appl. Phys.*.

[68] Rakic A. D., Djurisic A. B., Elazar J. M., Majewski M. L. (1998). Optical properties of metallic films for vertical-cavity optoelectronic devices. *Appl. Opt.*.

[69] Elton D. C. (2017). The origin of the Debye relaxation in liquid water and fitting the high frequency excess response. *Phys. Chem. Chem. Phys.*.

[70] Firouzi F., Sadrnezhaad S. K. (2023). Revisiting the experimental dielectric function datasets of gold in accordance with the Brendel-Bormann model. *J. Mod. Opt.*.

[71] De Sousa Meneses D., Gruener G., Malki M., Echegut P. (2005). Causal Voigt profile for modeling reflectivity spectra of glasses. *J. Non-Cryst. Solids*.

[72] Orosco J., Coimbra C. F. M. (2018). Optical response of thin amorphous films to infrared radiation. *Phys. Rev. B*.

[73] Nordebo S., Stumpf M. (2024). Time-domain constraints for passive materials: the Brendel-Bormann model revisited. *Phys. Rev. B*.

[74] Franta D., Vohanka J., Cermak M., Stenzel O., Ohlidal M. (2018). Universal dispersion model for characterization of thin films over wide spectral range. *Optical Characterization of Thin Solid Films*.

[75] Grosse C. (2014). A program for the fitting of Debye, Cole-Cole, Cole-Davidson, and Havriliak-Negami dispersions to dielectric data. *J. Colloid Interface Sci.*.

[76] Volkov A. S., Koposov G. D., Perfilev R. O., Tyagunin A. V. (2018). Analysis of experimental results by the Havriliak-Negami model in dielectric spectroscopy. *Opt. Spectrosc.*.

[77] Smith N. V. (2001). Classical generalization of the Drude formula for the optical conductivity. *Phys. Rev. B*.

[78] Wemple S. H., DiDomenico M. (1971). Behavior of the electronic dielectric constant in covalent and ionic materials. *Phys. Rev. B*.

[79] Tchebichef P. (1874). Sur les valeurs limites des integrales. *J. Math. Pure Appl.*.

[80] Prokopeva L. J., Borneman J. D., Kildishev A. V. (2011). Optical dispersion models for time-domain modeling of metal-dielectric nanostructures. *IEEE Trans. Magn.*.

[81] Prokopeva L. J., Henshaw W. D., Schwendeman D. W., Kildishev A. V., Werner D. H., Campbell S. D., Kang L. (2020). Time domain modeling with the generalized dispersive material model. *Nanoantennas and Plasmonics: Modelling, Design and Fabrication*.

[82] Angel J. B. (2019). A high-order accurate scheme for Maxwell’s equations with a generalized dispersive material model. *J. Comput. Phys.*.

[83] Banks J. W. (2020). A high-order accurate scheme for Maxwell’s equations with a generalized dispersive material (GDM) model and material interfaces. *J. Comput. Phys.*.

[84] Xia Q. (2022). High-order accurate schemes for Maxwell’s equations with nonlinear active media and material interfaces. *J. Comput. Phys.*.

[85] Hastings C. (1955). *Approximations for Digital Computers*.

[86] Fried B. D., Hedrick C. L., McCune J. (1968). Two-pole approximation for the plasma dispersion function. *Phys. Fluids*.

[87] Cody W. J., Paciorek K. A., Thacher H. C. (1970). Chebyshev approximations for Dawson’s integral. *Math. Comput.*.

[88] McCabe J. H. (1974). A continued fraction expansion, with a truncation error estimate, for Dawson’s integral. *Math. Comput.*.

[89] Hui A. K., Armstrong B. H., Wray A. A. (1978). Rapid computation of the Voigt and complex error functions. *J. Quant. Spectrosc. Radiat. Transf.*.

[90] Humlicek J. (1979). An efficient method for evaluation of the complex probability function: the Voigt function and its derivatives. *J. Quant. Spectrosc. Radiat. Transf.*.

[91] Humlicek J. (1982). Optimized computation of the Voigt and complex probability functions. *J. Quant. Spectrosc. Radiat. Transf.*.

[92] Martin P., Donoso G., Zamudio-Cristi J. (1980). A modified asymptotic Padé method. Application to multipole approximation for the plasma dispersion function Z. *J. Math. Phys.*.

[93] Martin P., Puerta J. (1981). Generalized Lorentzian approximations for the Voigt line shape. *Appl. Opt.*.

[94] Puerta J., Martin P. (1981). Three and four generalized Lorentzian approximations for the Voigt line shape. *Appl. Opt.*.

[95] Weideman J. A. C. (1994). Computation of the complex error function. *SIAM J. Numer. Anal.*.

[96] Lether F. G. (1997). Constrained near-minimax rational approximations to Dawson’s integral. *Appl. Math. Comput.*.

[97] Baalrud S. D. (2013). The incomplete plasma dispersion function: properties and application to waves in bounded plasmas. *Phys. Plasmas*.

[98] Abrarov S. M., Quine B. M. (2018). A rational approximation of the Dawson’s integral for efficient computation of the complex error function. *Appl. Math. Comput.*.

[99] Xie H. (2019). BO: a unified tool for plasma waves and instabilities analysis. *Comput. Phys. Commun.*.

[100] Alomar A. S. (2022). Application of the Martin-Donoso-Zamudio multipole approximation for generalized Faddeeva/Voigt broadening of model dielectric functions. *Thin Solid Films*.

[101] Alomar A. S. (2022). Impact of Faddeeva-Voigt broadening on line-shape analysis at critical points of dielectric functions. *AIP Adv.*.

[102] Xie H. (2024). Rapid computation of the plasma dispersion function: rational and multi-pole approximation, and improved accuracy. *AIP Adv*..

[103] Pfeifer M., Huynh D. N., Wegner G., Intravaia F., Peschel U., Busch K. (2024). Time-domain modeling of interband transitions in plasmonic systems. *Appl. Phys. B*.

[104] Moody G. (2015). Intrinsic homogeneous linewidth and broadening mechanisms of excitons in monolayer transition metal dichalcogenides. *Nat. Commun.*.

